# Heterogeneous Cu_*x*_O Nano-Skeletons from Waste Electronics for Enhanced Glucose Detection

**DOI:** 10.1007/s40820-024-01467-5

**Published:** 2024-07-18

**Authors:** Yexin Pan, Ruohan Yu, Yalong Jiang, Haosong Zhong, Qiaoyaxiao Yuan, Connie Kong Wai Lee, Rongliang Yang, Siyu Chen, Yi Chen, Wing Yan Poon, Mitch Guijun Li

**Affiliations:** 1 Center on Smart Manufacturing, Division of Integrative Systems and Design, The Hong Kong University of Science and Technology, Clear Water Bay, Kowloon, Hong Kong SAR 999077 People’s Republic of China; 2 The Sanya Science and Education Innovation Park of Wuhan University of Technology, Sanya, 572000 People’s Republic of China; 3 State Key Laboratory of New Textile Materials and Advanced Processing Technologies, Wuhan Textile University, Wuhan, 430200 People’s Republic of China

**Keywords:** Copper oxide, Electron 3D tomography, E-waste, Glucose detection, Electrochemical activation

## Abstract

Electronic waste (e-waste) and diabetes are global challenges to modern societies. However, solving these two challenges together has been challenging until now. Herein, we propose a laser-induced transfer method to fabricate portable glucose sensors by recycling copper from e-waste. We bring up a laser-induced full-automatic fabrication method for synthesizing continuous heterogeneous Cu_*x*_O (h-Cu_*x*_O) nano-skeletons electrode for glucose sensing, offering rapid (< 1 min), clean, air-compatible, and continuous fabrication, applicable to a wide range of Cu-containing substrates. Leveraging this approach, h-Cu_*x*_O nano-skeletons, with an inner core predominantly composed of Cu_2_O with lower oxygen content, juxtaposed with an outer layer rich in amorphous Cu_*x*_O (a-Cu_*x*_O) with higher oxygen content, are derived from discarded printed circuit boards. When employed in glucose detection, the h-Cu_*x*_O nano-skeletons undergo a structural evolution process, transitioning into rigid Cu_2_O@CuO nano-skeletons prompted by electrochemical activation. This transformation yields exceptional glucose-sensing performance (sensitivity: 9.893 mA mM^−1^ cm^−2^; detection limit: 0.34 μM), outperforming most previously reported glucose sensors. Density functional theory analysis elucidates that the heterogeneous structure facilitates gluconolactone desorption. This glucose detection device has also been downsized to optimize its scalability and portability for convenient integration into people’s everyday lives.

**Supplementary Information:**

The online version contains supplementary material available at 10.1007/s40820-024-01467-5.

## Highlights

- Novel laser-induced transfer method for fabricating glucose sensors from recycled e-waste copper, offering a sustainable and cost-effective solution.
- Unique heterogeneous Cu_*x*_O nano-skeletons derived from discarded printed circuit boards exhibiting exceptional glucose-sensing performance (sensitivity: 9.893 mA mM^−1^ cm^−2^, detection limit: 0.34 μM).
- Miniaturized glucose detection device, optimized for scalability and portability, revolutionizing diabetes management and patient care.

**Supplementary Information:**

The online version contains supplementary material available at 10.1007/s40820-024-01467-5.

## Graphical abstract

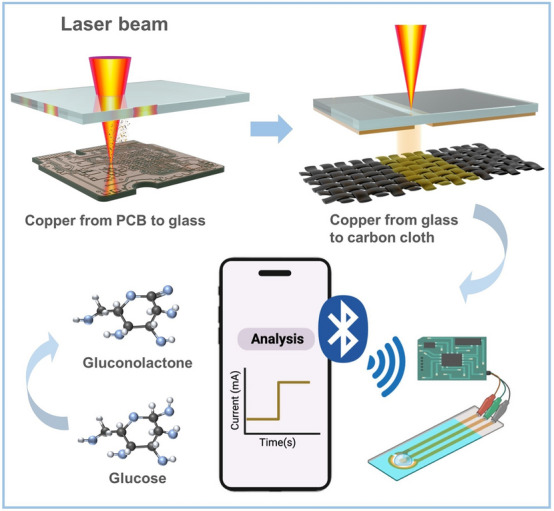

## Introduction

The growing use of electronic devices has made handling e-waste crucial, prompting a significant focus on developing effective methods for its disposal [1–3]. Recycling technologies for printed circuit boards (PCBs) can be generally categorized into physical and chemical methods [4, 5]. During physical recycling, the PCBs are mechanically dismantled and ground. Subsequently, the resulting broken PCBs are separated using a wind-shaking table or flotation separation technology based on differences in density, particle size, electrical conductivity, or magnetic conductivity [6, 7]. In chemical recycling, two standard methods are employed: hydrometallurgy, which yields sludge and acidic waste liquids, and thermometallurgy, which results in furans and dioxins [8–10]. One disadvantage of physical or chemical recycling methods is their potential for high cost and environmental pollution [11, 12]. Thermometallurgy, which involves subjecting the entire PCB to extreme heat, risks the volatilization and subsequent formation of hazardous byproducts, notably polybrominated dibenzo-p-dioxins, and dibenzofurans, from the brominated flame retardants ubiquitous in FR-4 substrates [8]. This necessitates stringent downstream gas treatment and poses a significantly higher ecological risk. Conversely, existing hydrometallurgy recycling techniques, which utilize leaching agents such as cyanide, thiourea, thiosulfate, and halides to dissolve and extract metallic components from PCBs selectively, generate substantial volumes of highly toxic wastewater containing cyanides, halides, or acids [10]. These effluents severely threaten soil and water resources if not properly treated.

Raele et al*.* introduced an efficient and environmentally friendly laser stripping method to remove the solder mask from PCBs, exposing the underlying copper [13]. However, such methods cannot recover the exposed copper. Laser technology shows promise not only for removing solder masks but also for metal transfer. Laser-induced back transfer (LIBT) is a technique that can transfer metal from the donor to the acceptor, which offers potential value for PCB metal recovery [14]. Laser-based metal recovery techniques offer a highly selective approach to PCB recycling, ablating and transferring only the targeted conductive layers (typically copper) and the overlying solder mask. This precise material removal minimizes interaction with the underlying FR-4 substrate. A localized extraction system effectively captures any airborne particulate matter (predominantly epoxy resin and pigment) generated during the laser ablation. This method is increasingly recognized for its comparatively low environmental impact, as it avoids the high-temperature processing inherent to traditional thermometallurgy methods [13].

Noninvasive glucose detection holds significant importance in managing diabetic patients [15–19]. Human sweat contains many essential biomarkers, including metabolites (glucose), electrolytes, amino acids, etc. [20]. Numerous papers have reported detecting glucose concentration in sweat using various electrode materials [21–23]. More attention has been paid to nonenzymatic glucose monitoring due to the susceptibility of enzymes to pH and temperature variations, their low stability, and high cost [24–26]. In nonenzymatic glucose sensing, copper oxide has emerged as a promising candidate owing to its favorable biocompatibility and high sensitivity to glucose [27, 28]. Various methodologies, including hydrothermal process [29], electrodeposition [30], calcination [31], and chemical reduction [32], have been used to get copper-based electrodes for glucose detection. Nonetheless, these approaches typically involve multiple procedural steps, prolonged waiting periods, and hazardous chemicals. To address these challenges, the laser-induced process offers an environmentally friendly, rapid, and scalable approach for fabricating copper-based electrodes, which can be adapted for various manufacturing techniques, including carbonization [33], oxidation [34], and deposition [35].

Herein, we present a laser-induced technique for producing continuous heterogeneous Cu_*x*_O (h-Cu_*x*_O) structures tailored for glucose sensing, offering rapid (< 1 min), clean, and air-compatible fabrication from waste electronics. Utilizing this method, we synthesized h-Cu_*x*_O nano-skeletons from PCBs. Such a structure comprises a continuous nano-skeleton, with an inner core predominantly composed of Cu_2_O with lower oxygen content, juxtaposed with an outer layer rich in amorphous Cu_*x*_O (a-Cu_*x*_O) with higher oxygen content. When employed in glucose detection, the h-Cu_*x*_O nano-skeletons undergo a structural evolution process, transitioning into rigid Cu_2_O@CuO nano-skeletons prompted by electrochemical activation. This transformation yields exceptional performance, with a sensitivity of 9.893 mA mM^−1^ cm^−2^ and a low detection limit of 0.34 μM, which outperforms most previously reported glucose sensors. Density functional theory (DFT) analysis clarifies the effectiveness of the heterogeneous structure lies in promoting gluconolactone desorption. Furthermore, we have miniaturized this glucose detection device to enhance its scalability and portability, facilitating the utilization of such a portable glucose detection device as telemedicine devices.

## Experimental Section

### Materials

PCBs were provided by Huaqiu Co., Ltd. Commercial CuO and Cu_2_O nanoparticles were purchased from Hongwu Newmaterial Co., Ltd. KOH and glucose were obtained from Fisher Chemical. Nafion solution, uric acid (UA), ascorbic acid (AA), and urea were obtained from Aladdin Reagent Co., Ltd. KCl was obtained from Sinopharm Chemical Reagent Co., Ltd. Lactate was supplied from Sigma-Aldrich. NaCl was supplied by Fuchen Chemical Reagent Co., Ltd. The mini electrochemical workstation based on the original circuit board design we provided was fabricated by Huaqiu Co., Ltd.

### Preparation of h-Cu_***x***_O Electrode

Heterogeneous Cu_*x*_O (h-Cu_*x*_O) preparation involves three steps: laser stripping, laser-induced backward transfer (LIBT), and laser-induced forward transfer (LIFT). The laser source is a nanosecond Nd: YAG laser, and the wavelength is 1064 nm. It is important to note that all laser machines are equipped with a ventilation system to prevent generating hazardous gas and chemicals during laser operation. During the laser stripping process, the varnish on the PCB was removed using the following parameters: 1000 mm s^−1^ in speed, 40 kHz in repetition frequency, and 40 ns in pulse. After laser stripping, the fresh copper on the PCB was left exposed to ambient conditions. The next step was applying the LIBT process to recycle the copper from the PCB (donor) to the plain glass (acceptor). This step focuses on the efficiency of copper recycling. The mass reduction of the PCB and the corresponding mass increase of the glass sheet after each LIBT process were recorded, as shown in Table S1. The average recycling efficiency is 82.51%. The laser parameters are 230 mm s^−1^ in speed and 7 kHz in repetition frequency. Some image processing techniques were used to filter out copper areas in the PCB board by simply taking one image. The PCB image underwent an initial transformation into a grayscale representation, as shown in Fig. S1. Thresholding techniques, utilizing the grayscale intensity values, are then employed to effectively segment copper regions from other areas present in the image. Those recognized copper coordinates can be then transferred into consecutive paths and exported as laser printing files to transfer the copper out. Therefore, aided by this image recognition technique, the dimensions (length and width) of the conductor area do not pose a limitation to the laser transfer process. The majority of commercially available PCBs utilize a 35 μm copper layer, which is the chosen substrate for this work. Only a limited number of applications with specific requirements, such as high-frequency performance or cost reduction, opt for thinner 18 µm copper PCBs. Conversely, high current capacity applications may employ PCBs with thicker 70 µm copper layers. Experimental findings demonstrate that a PCB with a 35 µm copper layer can reliably undergo approximately 8 LIBT cycles onto glass substrates. This observation suggests that the PCBs with 18 µm copper layers could withstand 4 LIBT transfer cycles, while those with 70 µm copper layers could endure up to 16 cycles. In addition, PCB aging can potentially affect the internal copper. However, laser transfer offers the advantage of selective area targeting. In cases where copper corrosion has occurred, these specific regions can be avoided during the transfer process. Finally, the LIFT process was employed to transfer the copper compound from the glass to the carbon cloth surface. This step focuses on the preparation efficiency of nano-catalysts. We observed that two transfers were able to transfer the majority of the copper from the glass slides to the carbon cloth. However, multiple transfers resulted in alterations to the product’s properties, thereby impacting its glucose-sensing performance. Therefore, a single transfer process was employed in this study. The speed of laser parameters is 400 mm s^−1^, and the frequency was set as 100, 200, 300, 400, 500 kHz, and continuous-wave (CW) mode. At last, the carbon cloth with copper compounds was utilized as the working electrode.

### Preparation of Commercial Cu_2_O and CuO Electrode

The commercial Cu_2_O or CuO electrode was prepared as follows. The first step involved dispersing commercial Cu_2_O or CuO nanoparticles in a mixed solution of 400 μL ethanol, 550 μL deionized water, and 50 μL 5% Nafion. Next, the obtained solution was sonicated for 30 min to make the commercial Cu_2_O or CuO nanoparticles disperse uniformly in the solution. Then, 100 μL dispersed solution was dipped in 1 × 1 cm^2^ carbon cloth and dried in the ambient environment for 6 h. Lastly, the electrode obtained can be applied to electrochemical measurements.

### Surface Characterization

X-ray diffraction (XRD) patterns were acquired by PANalytical X’pert Pro equipment. X-ray photoelectron spectroscopy (XPS) databases were obtained with Kratos Axis Ultra DLD. The Raman spectra were investigated using InVia-Renishaw equipment. The microstructures of the samples were characterized by JEOL-6700F scanning electron microscope and Titan Themis3 G2 300 transmission electron microscope.

### Electrochemical Measurements

The PARSTAT 3000A-DX electrochemical workstation took all electrochemical measurements via the standard three-electrode system. Ag/AgCl and platinum (Pt) plate electrodes were applied as the reference and counter electrodes. The cyclic voltammetry (CV) measurement was tested from 0 to 0.7 V versus Ag/AgCl in 0.1 M KOH. The chronoamperometry (CA) measurement was taken under constant magnetic stirring at 0.6 V versus Ag/AgCl, dropping a specific glucose solution every 50 s.

### Theoretical Calculation

DFT calculation was performed to investigate the glucose-sensing mechanism on different electrode surfaces with the Vienna ab initio Simulation Package (VASP) [36, 37]. In the DFT calculations, the projector augmented wave (PAW) method was employed in conjunction with the generalized gradient approximation (GGA) using the Perdew–Burke–Ernzerhof (PBE) functional [38, 39]. To investigate the binding energy of glucose molecules to substrates (Cu_2_O-100 and CuO-100), we created a 2 × 2 × 1 supercell slab model with three atomic layers that simulated the (100) facet of Cu_2_O and CuO. To demonstrate the effect of heterostructure on glucose adsorption/catalysis, a slab model of a 2 × 2 × 1 supercell with two atomic layers, simulating the (110) facet of Cu_2_O, was added to the Cu_2_O slab (Cu_2_O/CuO heterostructure). We included a vacuum slab of approximately 15 Å between the surface slabs for all models. The kinetic cutoff energy was 500 eV for the plane-wave expansion, and the calculations also considered spin polarization. The convergence criteria for geometry optimization were set as the energy and force, with a convergence threshold of 1.0 × 10^−6^ eV atom^−1^ and 0.05 eV Å^−1^. We used a 4 × 4 × 4 k-point mesh for Brillouin-zone sampling to ensure total energy convergence. The free energy change was the following:

1 $$\Delta G\, = \,\Delta E\, + \,\Delta G_{{{\text{ZPE}}}} \,{-}\,T\Delta S\,{-}\,neU$$

Δ*E* refers to the energy change by the DFT calculation, Δ*G*_ZPE_ refers to the zero-point energy, and Δ*S* refers to the entropy correction.

The following formula determined the adsorption energy (Δ*E*_glucose*_):

2 $$\Delta E_{{{\text{glucose}}^{*} }} \, = \,E_{{\text{surf + glucose}}} \, - \,E_{{{\text{surf}}}} \, - \,E_{{{\text{glucose}}}}$$

*E*_surf+glucose_ refers to the total energy of glucose adsorbed on different surfaces, *E*_surf_ and *E*_glucose_ are the energy of pure surfaces and a single glucose molecule.

## Results and Discussion

### Automatic Fabrication for the Heterogeneous Cu_***x***_O Glucose Detection Electrode

**Fig. 1:**
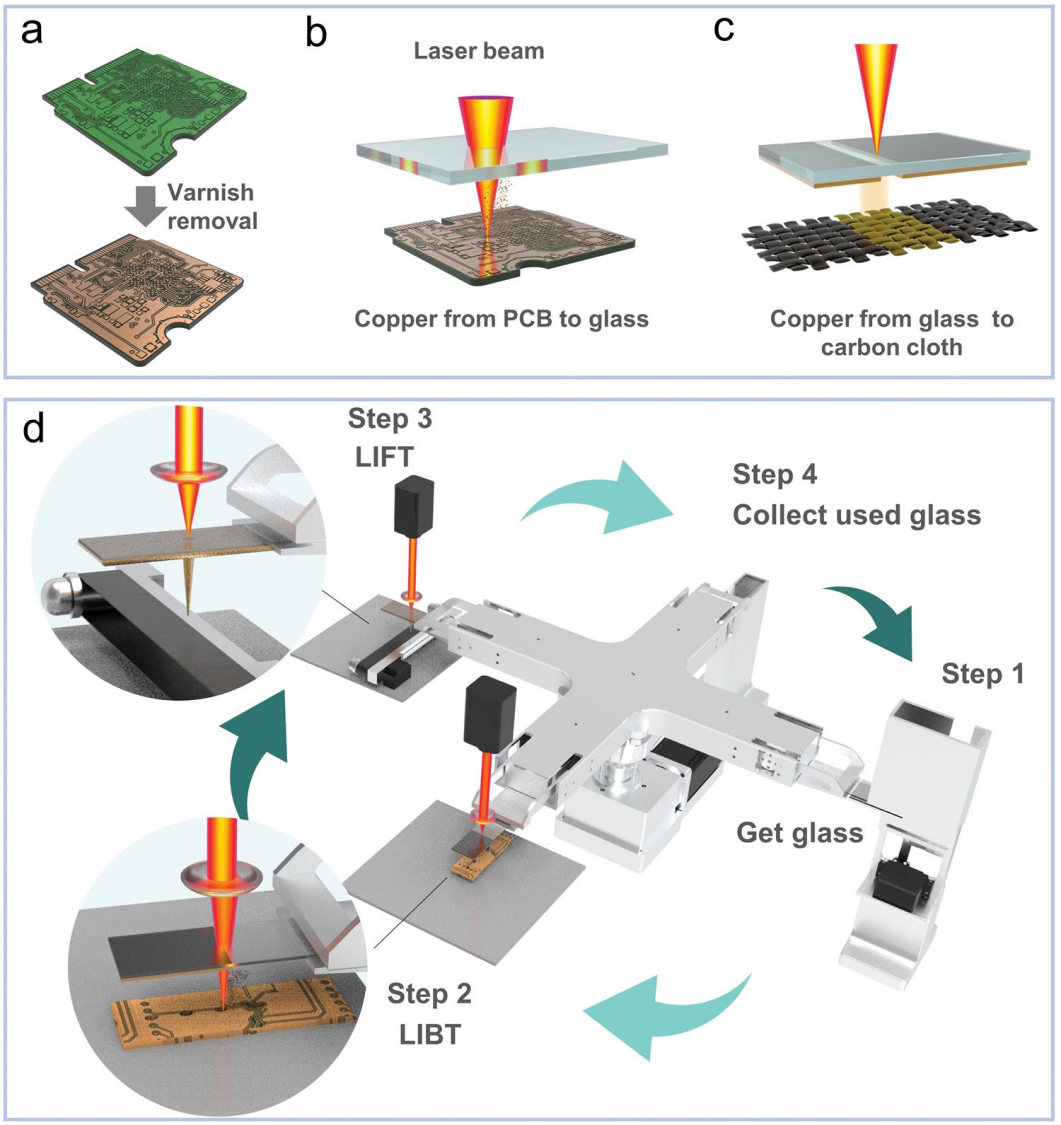
Design of automatic fabrication for the h-Cu_*x*_O glucose detection electrode. **a** PCB before and after varnish removal. **b** LIBT process. **c** LIFT process. **d** Automatic device to fabricate h-Cu_*x*_O electrode

A rapid, low-cost, environmentally friendly, and scalable method was used to fabricate h-Cu_*x*_O electrodes by a laser-induced transfer process from waste PCBs (Fig. 1). Figure 1a shows the original PCB with a layer of green varnish on the surface. This varnish was stripped by laser writing before the laser transfer procedure to get higher-purity copper. The optical images of the PCB with and without green varnish are displayed in Fig. S2. The green varnish of PCBs is thoroughly removed, leaving the underneath copper fully exposed as the substrate for the subsequent laser transfer process. Next, the copper from the PCBs was transferred to the plain glass sheet by LIBT process (Fig. 1b). Then, the copper on the glass sheet was transferred to the carbon cloth substrate by a LIFT process (Fig. 1c). Based on the laser transfer method, a fully automatic fabrication system was developed, which allows a continuous electrode fabrication process once laser fabricating parameters are established (Figs. 1d, S3 and Video S1). The step 1 and step 4 can be finished in 3 s, respectively. And the time to complete step 2 and step 3 is around 20 and 25 s, respectively. So the time for completing all 4 steps is less than 1 min.

The LIBT process is captured in Fig. S4 to better understand this process. The laser beam knocked down the PCB surface, and then, vaporized particles were generated, and white plasma was observed in this process. Then, the copper from the PCB was deposited on the glass sheet, forming a mixture of Cu and Cu_2_O (Figs. S5, S6). The LIFT process was also recorded, as shown in Fig. S7. The laser beam penetrated the glass sheet to the interface between the glass sheet and the copper layer. The affected area was in a high-temperature condition, and the copper was ejected into the carbon cloth by the vapor and plasma. In the LIFT process, the frequency of the laser beam significantly impacts the results, so a series of samples using different frequency parameters were studied. It is evident that with the increasing laser frequency, the ratio of Cu_2_O:Cu will also increase until the Cu almost disappears when the laser beam is working in CW mode (regarded as laser frequency is infinitely high, Fig. S8). The kinetic mechanism in the LIFT process is studied in Table S2 and Fig. S9. It is found that the laser pulse with a lower frequency will lead to a higher surface temperature of the donor, contributing to a more intense micro-detonation and a more rapid transfer process. The proportion of actual laser transfer time positively correlates with the laser frequency under a single laser pulse irradiation, as shown in Fig. S10. Generally, the longer the transfer time, the greater the likelihood of oxidation occurring on copper particles. Fig. S11 shows the CV results of these samples in 0.1 M KOH containing 0.5 mM glucose. The peak current density and laser frequency parameters have a positive relationship, indicating that more Cu_2_O with higher laser frequency leads to improved glucose-sensing performance, and the sample (h-Cu_*x*_O) obtained by CW mode shows the highest glucose detection ability.

### Chemical State and Electron Microscopy Characterization of Heterogeneous Cu_x_O

**Fig. 2:**
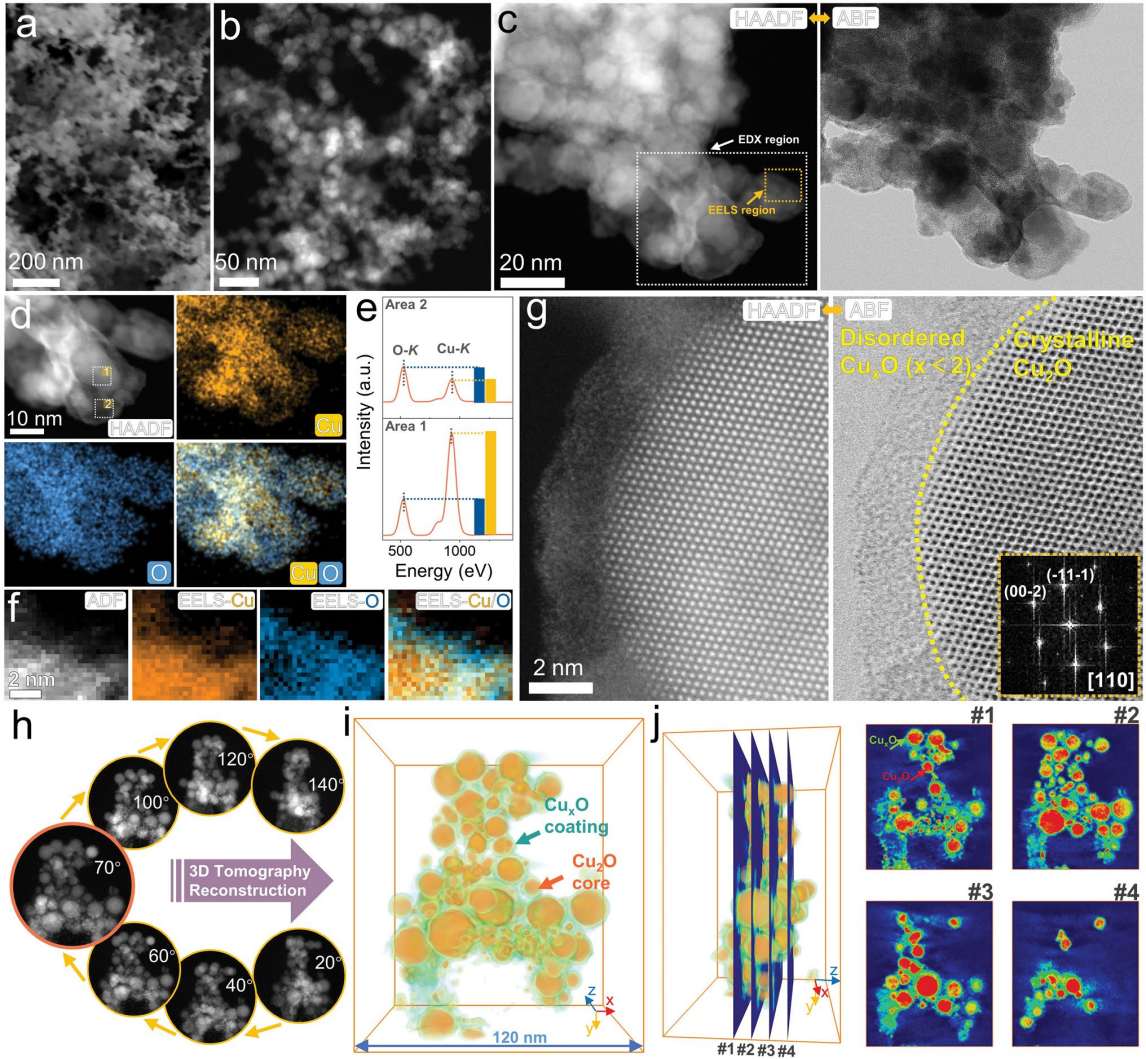
Structure and morphology characterization of h-Cu_*x*_O sample. **a** SEM image. **b** TEM image. **c** HAADF-STEM image and corresponding ABF-STEM image. **d** HAADF-STEM image and corresponding EDX mapping images in c. **e** EDX spectra from areas 1 and 2 in **d**. **f** ADF image and corresponding EELS mapping of Cu, O, and color mix in c. **g** High-resolution HAADF-STEM image and ABF-STEM image with corresponding FFT pattern. **h** Representative HAADF-STEM images at different rotation angles. **i** Reconstructed the h-Cu_*x*_O model. **j** Representative orthoslices

Fig. 2 illustrates the morphology and structure characterization of the h-Cu_*x*_O sample. The scanning electron microscopy (SEM, Fig. 2a) displays a three-dimensional continuous structure comprised of bridged nanoparticles. Transmission electron microscopy (TEM, Fig. 2b) reveals that the nanoparticles have a diameter of approximately 20 nm. High-angle annual dark field-scanning transmission electron microscopy (HAADF-STEM, Fig. 2c) demonstrates an encapsulation structure where the contrast of the inner part is brighter than the outer layer, and the annual bright-field (ABF) image also confirms such an encapsulation structure. The corresponding energy-dispersive X-ray spectroscopy (EDX) and electron energy loss spectroscopy (EELS) are shown in Figs. 2d–f. The EDX mapping (Fig. 2d) reveals that the Cu signal in the interior is stronger compared to the exterior, and the O signal within the interior exhibits a similar intensity to that of the exterior, which is also supported by the EDX analysis (Fig. 2e). The EELS mapping results (Fig. 2f) provide additional evidence of higher Cu concentration inside the encapsulation, resulting in a brighter interior compared to the exterior in this structure. This phenomenon is attributed to STEM’s contrast being proportional to atomic mass. Further exploration of the nano-structure is conducted using atomic-resolution HAADF-STEM and ABF-STEM, as shown in Fig. 2g. The bright contrast and ordered atomic lattice arrangement of the inner core correspond to the crystal lattice of Cu_2_O. Meanwhile, the darker contrast and disordered atomic arrangement of the outer coating layer correspond to a-Cu_*x*_O. The fast Fourier transform (FFT) image corresponding to the HAADF-STEM image from the inner core (inset in Fig. 2g) proves the [1 1 0] orientation of Cu_2_O.

The 3-dimensional tomography reconstruction further proves the encapsulation structure. We collected 72 HAADF-STEM images at 2-degree intervals for tomography reconstruction, as shown in Fig. 2h and Video S2. The reconstructed model depicted in Fig. 2i closely mirrors the shape observed in the HAADF image captured at 70°, as highlighted within an orange frame in Fig. 2h. The bounding box has dimensions of 120 nm (length, *x*), 139 nm (height, *y*), and 80 nm (width, *z*), respectively. Volume segmentation based on contrast is employed to extract the volume of the Cu_2_O core, given its higher contrast in the HAADF image. The a-Cu_*x*_O coating was observed to be continuous, but the extracted Cu_2_O cores were mostly isolated. To provide a clear visual representation, the volume of the a-Cu_*x*_O coating was converted to a semi-transparent green color, forming a color-mixed model of h-Cu_*x*_O (Fig. 2i). This model identifies a uniform 3D complete encapsulation structure, as seen in the three-view drawing (Fig. S12) and orthoslices (Fig. 2j). XPS and Auger spectra further investigated the chemical state of the h-Cu_*x*_O sample. Fig. S13 shows the detailed Cu 2*p* and Cu LMM Auger scan of the h-Cu_*x*_O. The Cu 2*p*_3/2_ spectrum indicates 2 components of Cu^2+^ at 933.9 eV and Cu^+^ at 931.8 eV [40]. And the satellite peak also reveals the presence of Cu^2+^ in the h-Cu_*x*_O sample [41]. The central peak of the Cu Auger spectrum is located at around 570.6 eV, which also implies the existence of Cu^+^ in the h-Cu_*x*_O sample [42].

### Electrochemical Glucose Detection Performance

This study compares the h-Cu_*x*_O, commercial Cu_2_O, and CuO nanoparticles as the working electrodes for glucose detection. The diameter of commercial Cu_2_O nanoparticles and CuO nanoparticles is about 25 nm (Fig. S14), comparable to the size of Cu_2_O in the h-Cu_*x*_O sample. During the electrochemical measurements, it was found that the electrochemical performance of the h-Cu_*x*_O would be improved after the electrochemical activation process (CA test at 0.6 V versus Ag/AgCl for 30 min). After electrochemical activation, these samples are named h-Cu_*x*_O-EA, commercial Cu_2_O-EA, and CuO-EA. CV measurement was studied to investigate the electrochemical properties of the electrode for detecting glucose. In Fig. S15, the CV results of the h-Cu_*x*_O, commercial Cu_2_O, and CuO electrodes in the 0.1 M KOH were measured at 50 mV s^−1^ before and after electrochemical activation. Before electrochemical activation, the commercial CuO electrode shows the lowest current density. The commercial Cu_2_O electrode has a larger anodic current than the h-Cu_*x*_O electrode and a similar cathodic current density to the h-Cu_*x*_O electrode. However, the situation changed after the electrochemical activation. Electrochemical activation significantly enhanced the current density of the h-Cu_*x*_O electrode, as observed. In contrast, the current density of commercial Cu_2_O and CuO electrodes had no noticeable change after electrochemical activation, which implies that the electrochemical activation process may only raise the amount of possible catalytic active sites for the h-Cu_*x*_O electrode among these three electrodes.

**Fig. 3:**
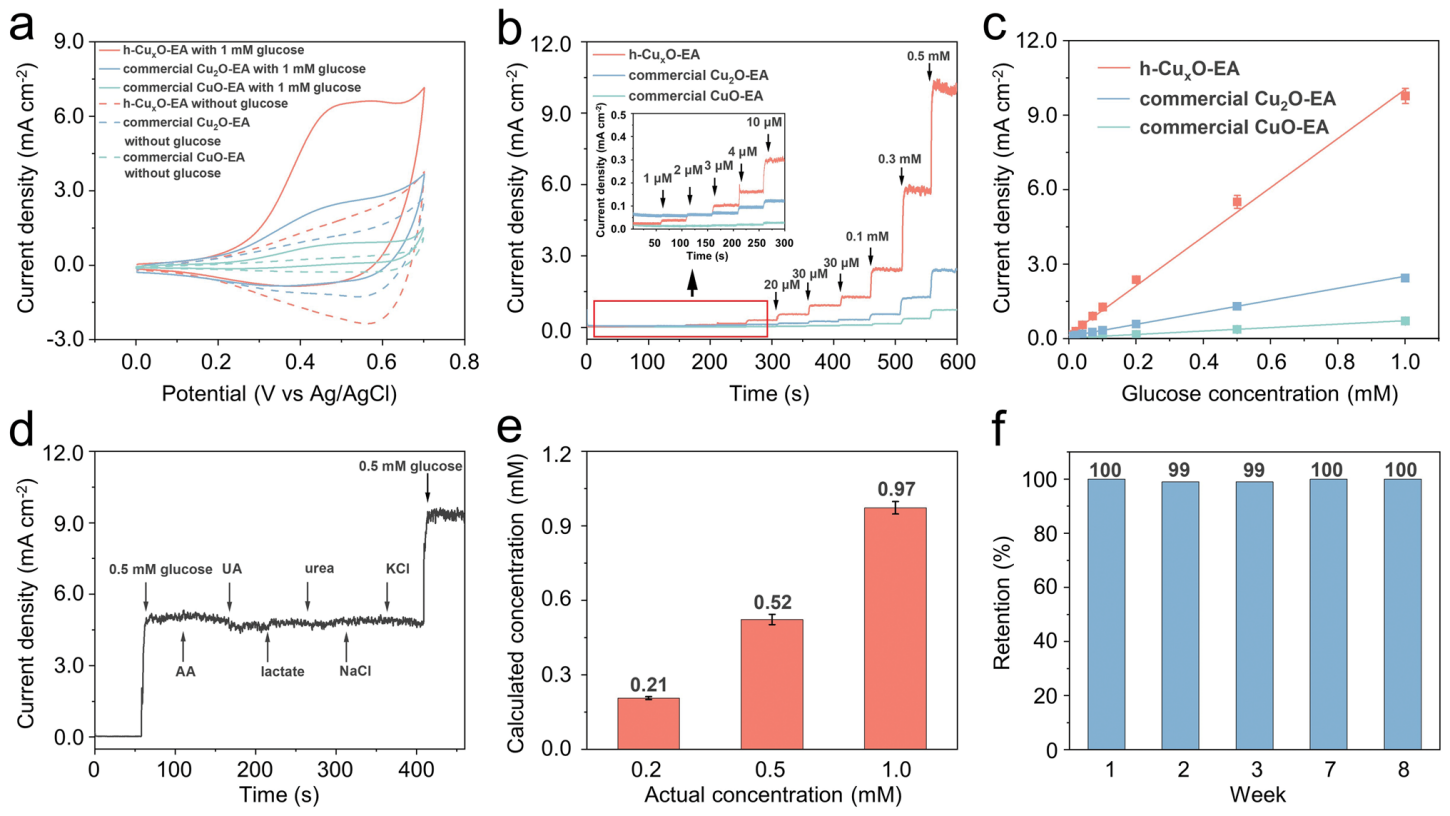
Glucose detection performance of the h-Cu_*x*_O-EA, commercial Cu_2_O-EA, and commercial CuO-EA electrodes. **a** CV curves of the h-Cu_*x*_O-EA, commercial Cu_2_O-EA, and commercial CuO-EA without glucose and within 1 mM glucose. **b** Amperometric responses of h-Cu_*x*_O-EA, commercial Cu_2_O-EA, and commercial CuO-EA electrodes to glucose. **c** The corresponding linear fitting curves of amperometric response with a different glucose concentration of h-Cu_*x*_O-EA, commercial Cu_2_O-EA, and commercial CuO-EA electrodes. **d** Amperometric response of h-Cu_*x*_O-EA with the addition of glucose, AA, UA, lactate, urea, NaCl, KCl, and again glucose. **e** The relationship of calculated glucose concentration with actual glucose concentration in the artificial sweat. **f** The stability of h-Cu_*x*_O-EA for 8 weeks

The redox reaction on the working electrodes in the 0.1 M KOH was studied as shown in Fig. 3a, which shows the reaction from 0 to 0.7 V versus Ag/AgCl without glucose and with 1 mM glucose at 50 mV s^−1^. When adding 1 mM glucose, all the h-Cu_*x*_O-EA, commercial Cu_2_O-EA, and CuO-EA electrodes present broad oxidation peaks because of the glucose oxidation. However, the current response of the h-Cu_*x*_O-EA electrode is higher than the commercial Cu_2_O-EA and CuO-EA electrodes, indicating that the h-Cu_*x*_O-EA electrode has the best ability to catalyze glucose oxidation. The possible mechanism underlying glucose sensing by the Cu-based electrode toward glucose detection can be illustrated as follows: first, Cu_2_O (Cu^I^) is electrochemically oxidized to CuO (Cu^II^) at roughly 0.4 V versus Ag/AgCl [43]; second, CuO is electrochemical oxidized to Cu(OH)_4_^−^ or CuOOH (Cu^III^) at approximately 0.55 V versus Ag/AgCl [44]; third, the CuOOH or Cu(OH)_4_^–^ catalyzes glucose oxidation to generate gluconolactone which increases anodic current [30]. The chemical reactions in this process are shown as follows:

3 $${\text{2OH}}^{ - } {\text{ + Cu}}_{{2}} {\text{O }} \to {\text{ 2CuO + H}}_{{2}} {\text{O + 2e}}^{ - }$$

4 $${\text{OH}}^{ - } {\text{ + CuO }} \to {\text{ Cu}}\left( {{\text{OH}}} \right)_{{4}}^{ - } {\text{ + e}}^{ - } {\text{ or OH}}^{ - } {\text{ + CuO }} \to {\text{ CuOOH + e}}^{ - }$$

5 $${\text{Glucose + Cu}}\left( {{\text{III}}} \right){ } \to {\text{ Gluconolactone + CuO + OH}}^{ - }$$

The cathodic peak at around 0.57 V for h-Cu_*x*_O-EA, commercial Cu_2_O-EA, and CuO-EA in the CV curve without glucose disappeared after adding 1 mM glucose. The reason is that this peak represents the conversion from Cu(III) to Cu(II). However, the Cu(III) would be depleted when detecting glucose (Eq. 5) [45]. The cathodic peak current of h-Cu_*x*_O-EA is higher than the commercial Cu_2_O-EA and CuO-EA, which suggests the h-Cu_*x*_O-EA has more Cu(III), thus showing better glucose detection performance. In Fig. S16a, the electrochemical reaction kinetics of glucose oxidation reaction in the h-Cu_*x*_O-EA sample containing 1 mM glucose was studied by performing CV at different scan rates (10–80 mV s^−1^). The glucose oxidation current grows with increasing scan rates. In Fig. S16b, the current generated from glucose oxidation changes in proportion to the rate of scanning (*R*^2^ = 0.993), which indicates the glucose oxidation procedure is a surface-absorption-controlled process.

The detection potential was investigated to enhance the glucose detection capability of the h-Cu_*x*_O-EA sample. Fig. S17 displays the amperometric response of the h-Cu_*x*_O-EA to continuously dropping 0.1 mM glucose solution in 0.1 M KOH at various potentials. By applying 4 different potentials (0.50, 0.55, 0.60, and 0.65 V) around the oxidation peak for the h-Cu_*x*_O-EA electrode, the CA measurement suggests that the best glucose-sensing performance appears at 0.60 V. Consequently, the working potential at 0.60 V was chosen for the subsequent CA tests. Fig. 3b demonstrates the current response of the h-Cu_*x*_O-EA, commercial Cu_2_O-EA, and CuO-EA electrodes toward the glucose in 0.1 M KOH electrolyte at 0.6 V. The glucose concentration is incrementally increased from 1 μM to 1 mM through successive dropwise additions. It is observed that a slight change of 1 μM in glucose concentration causes a significant current change in the h-Cu_*x*_O-EA electrode. However, the current response for commercial Cu_2_O-EA and CuO-EA electrodes is not apparent after adding 1 μM glucose to the electrolyte, which is in accord with the CV curves, implying that the h-Cu_*x*_O-EA electrode owns the best electrocatalytic performance. Fig. S18 demonstrates that among various pH electrolytes tested, the h-Cu_*x*_O-EA electrode exhibits optimal glucose detection performance at pH13, corresponding to the 0.1 M KOH electrolyte employed in this study.

Fig. 3c shows the calibration plot that correlates the measured current values with their respective glucose concentrations. The h-Cu_*x*_O-EA electrode displays the most pronounced sensitivity among the evaluated electrodes, with a value of 9.893 mA mM^−1^ cm^−2^ (*R*^2^ = 0.996). In comparison, the commercial Cu_2_O-EA and CuO-EA present lower sensitivity of 2.423 mA mM^−1^ cm^−2^ (*R*^2^ = 0.999), and 0.695 mA mM^−1^ cm^−2^ (*R*^2^ = 0.999), respectively. The limit of detection (LOD) for the h-Cu_*x*_O-EA electrode is determined to be 0.34 μM, calculated by LOD = 3*N*/*S* (*N* represents the standard deviation of ten blank currents, and *S* represents sensitivity). Table S3 shows that the h-Cu_*x*_O-EA electrode has a faster preparation time (< 1 min) and superior glucose detection performance compared to most reported glucose-sensing electrodes. The current response comparison of h-Cu_*x*_O-EA and h-Cu_*x*_O, commercial Cu_2_O-EA and commercial Cu_2_O, commercial CuO-EA and commercial CuO electrodes were also investigated, as shown in Fig. S19. The electrochemical activation process could significantly enhance the glucose detection capability of the h-Cu_*x*_O sample, and the sensitivity has been improved by 90%. Still, the sensitivity of commercial Cu_2_O and CuO has slightly improved after the electrochemical activation process. Fig. S20 shows the amperometric response to 1 mM glucose of the h-Cu_*x*_O-EA electrode, and the response time is 2 s, indicating the fast electron transfer [46].

The anti-interference capability of the h-Cu_*x*_O-EA was performed in the CA measurement by adding other substances commonly found in sweat, including AA, UA, lactate, urea, KCl, and NaCl. Fig. 3d displays the amperometric response of the h-Cu_*x*_O-EA to 0.5 mM glucose and 0.02 mM AA, 0.05 mM UA, 5 mM lactate, 5 mM urea, 30 mM NaCl, and 5 mM KCl. The h-Cu_*x*_O-EA electrode demonstrates the most outstanding amperometric response to glucose, and the current response to other interferential materials could be negligible, indicating excellent anti-interference capability. We also investigated the effectiveness of the h-Cu_*x*_O-EA electrode in detecting glucose concentration in artificial sweat, as shown in Fig. 3e. The artificial sweat is prepared by 0.02 mM AA, 5 mM lactate, 0.05 mM UA, 5 mM urea, 30 mM NaCl, and 5 mM KCl. Three kinds of artificial sweat with different glucose concentrations were tested, and the calculated glucose concentration was very close to the actual glucose concentration. The long-term durability of the h-Cu_*x*_O-EA was investigated by assessing the amperometric response toward 1 mM glucose at different intervals. Figs. 3f and S21 show that the h-Cu_*x*_O-EA electrode could achieve nearly 100% current retention after 8 weeks, indicating the excellent long-period stability of the h-Cu_*x*_O-EA electrode.

### Revealed Post-reaction Heterogeneous Structure and Active Sites

As mentioned above, only the performance of h-Cu_*x*_O improved after electrochemical activation. Thus, the structure evolution of this “activation” was carefully characterized and correlated with the electrochemical performance improvements. The surface structure and chemical characteristics of the electrode were characterized to investigate the electrochemical activation process. The morphology of h-Cu_*x*_O-EA shrinks little during the electrochemical activation process, as observed in the SEM image (Fig. S22). Figure S23 exhibits the TEM image of h-Cu_*x*_O-EA, indicating that the structure of h-Cu_*x*_O-EA stays roughly the same as the previous h-Cu_*x*_O sample. Figure S24 presents the HAADF-STEM and corresponding EDX elemental mapping of h-Cu_*x*_O-EA. It is observed that the copper and oxygen components are roughly distributed throughout the whole structure.

**Fig. 4:**
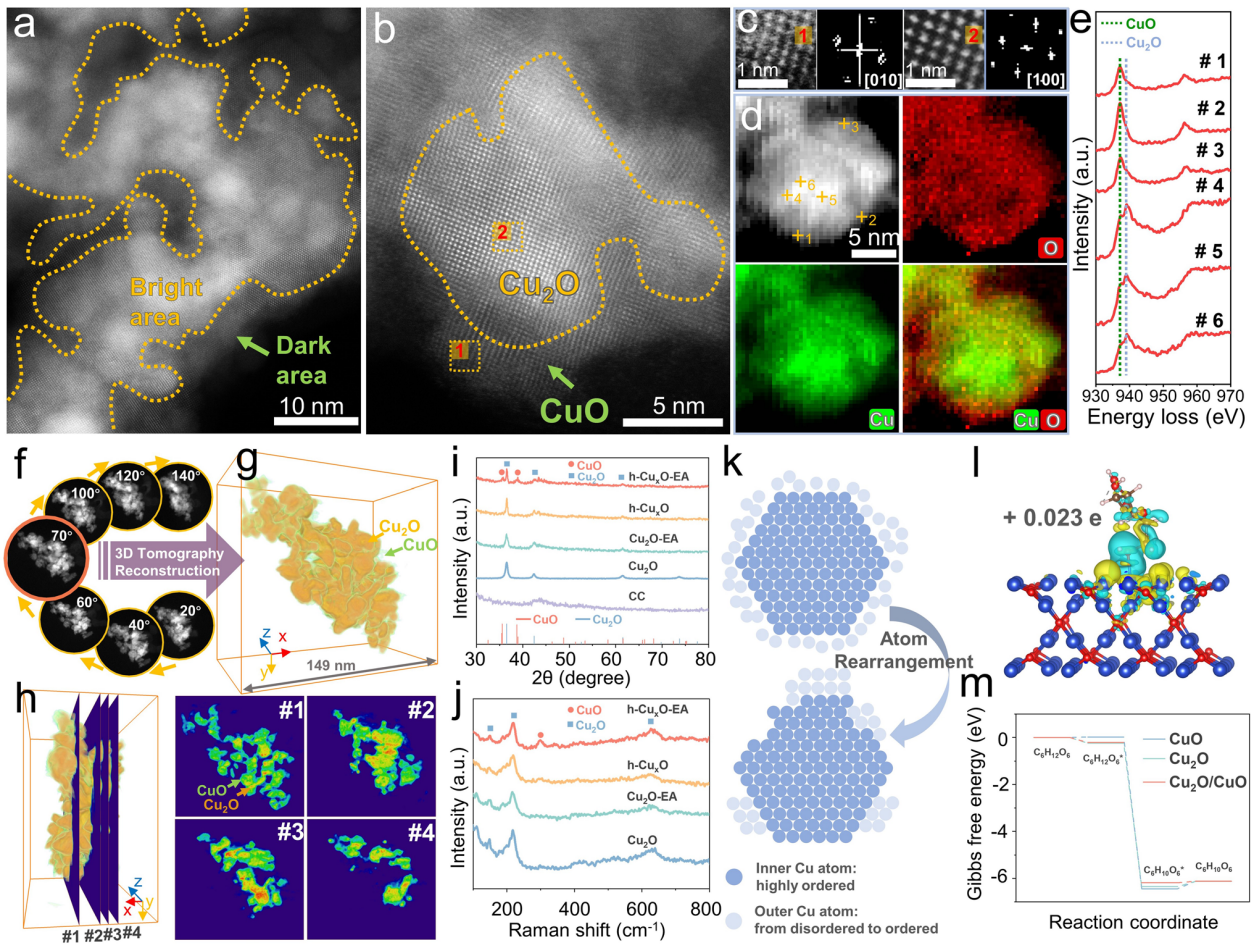
Revealed post-reaction heterogeneous structure and active sites. **a** HAADF-STEM image and **b** high-resolution HAADF-STEM image of h-Cu_*x*_O-EA. **c** Magnified HAADF image of area 1 and area 2 in b and corresponding FFT. **d** ADF image and corresponding EELS mapping of Cu and O elements of h-Cu_*x*_O-EA. **e** EELS spectra of Cu-L_2,3_ edge from 6 selected areas marked in d. **f** Representative HAADF-STEM images at different rotation angles of h-Cu_*x*_O-EA. **g** Reconstructed model and **h** representative orthoslices of the h-Cu_*x*_O-EA model. **i** XRD of h-Cu_*x*_O-EA, Cu_*x*_O, Cu_2_O-EA, Cu_2_O and laser-treated carbon cloth samples. **j** Raman spectra of h-Cu_*x*_O-EA, h-Cu_*x*_O, Cu_2_O-EA, and Cu_2_O samples. **k** Schematic diagram of atom rearrangement during the electrochemical activation process. **l** Calculated differential charge density of glucose absorbed on Cu_2_O/CuO heterojunction. **m** Free energy profiles of glucose oxidation reaction on CuO, Cu_2_O, and Cu_2_O/CuO models

In Figure 4a, the HAADF-STEM image exhibits that the h-Cu_*x*_O-EA sample surface contrasts with a bright inner part and a continuous thin dark coating layer. To further study the heterogeneous property of the h-Cu_*x*_O-EA sample, the atomic-resolution HAADF-STEM is investigated in Figure 4b. It is observed that the amorphous layer in the outer layer disappears compared with the h-Cu_*x*_O sample, and the surface is composed of different contrast areas. Figure 4c displays the magnified image of the dark area (area 1) and bright area (area 2) from Fig. 4b. The corresponding FFT patterns of areas 1 and 2 are determined to be the [0 1 0] orientation of CuO and [1 0 0] orientation of Cu_2_O, indicating the dark area corresponds to the CuO. In contrast, the bright area corresponds to the Cu_2_O, further proving heterojunction formation in the h-Cu_*x*_O-EA sample. The EELS mapping is illustrated in Fig. 4d, which reveals that the distribution of Cu and O are not uniform: the outer layer has more O distribution while the inner part has more Cu distribution. The heterogeneous property of h-Cu_*x*_O-EA is further studied by the EELS point scan as presented in Fig. 4e. 6 typical points are chosen from the marked points in Fig. 4d, which display the Cu-L_2,3_ edges. An energy gap of approximately 20 eV separates the L_2_ and L_3_ edges due to the 2*p* core hole’s spin–orbit interaction. The central peak of the L_3_ edge is situated at 937.1 eV, corresponding to CuO, and 939.3 eV, corresponding to Cu_2_O [47]. It is evident that spectra from points 1, 2, and 3 of the sample surface show mainly CuO distribution. The spectra from points 4, 5, and 6 of the inner part of the sample show the coexistence of CuO and Cu_2_O distributions, indicating the inhomogeneous distribution of these 2 phases in the whole structure.

The 3-dimensional tomography reconstruction characterization is studied in Fig. 4f and Video S3, constructed by 72 HAADF-STEM photographs at 2-degree intervals. Figure 4g shows the reconstructed model corresponding to the picture with an orange frame in Fig. 4f taken at 70°. The bounding box’s length, height, and width (*x*, *y*, *zoxy_insert_end*) are 149, 139, and 87 nm, respectively. The orange and green volumes correspond to the high and low contrast in the HAADF image, respectively. So, the volume in orange presents Cu_2_O, and the volume in green presents CuO. It is observed that the whole structure of h-Cu_*x*_O-EA has minor deformation. However, the encapsulation structure is still maintained compared with the h-Cu_*x*_O sample. The comparison of the reconstructed model between h-Cu_*x*_O and h-Cu_*x*_O-EA is shown in Fig. S25, and along with the previous STEM results, it is concluded that the a-Cu_*x*_O in the h-Cu_*x*_O sample forms the crystal CuO layer in the h-Cu_*x*_O-EA sample. To get a precise observation of the inner structure of the h-Cu_*x*_O-EA electrode, the orthoslices manipulation is applied (Fig. 4h), and it is evident that the interior and exterior have different contrasts. The XRD and Raman characterizations assisted in proving the crystal CuO formation in the h-Cu_*x*_O-EA sample, especially noting that the commercial Cu_2_O sample kept a stable crystal structure.

Figure 4k demonstrates the possible mechanism of the h-Cu_*x*_O sample during the electrochemical activation process. Initially, the a-Cu_*x*_O coating layer tightly wraps the Cu_2_O nanoparticles, and such a structure is stable in the ambient environment. However, the amorphous phase is more accessible for self-reconstruction than the crystalline counterpart under the oxidation potential [48]. Then, the electrochemical activation process will cause an atomic rearrangement for the a-Cu_*x*_O. The characterization of the commercial Cu_2_O nanoparticles before and after electrochemical activation suggests that the crystal structure of Cu_2_O nanoparticles would remain stable during the electrochemical activation process. The difference between the h-Cu_*x*_O and commercial Cu_2_O nanoparticles is introducing the a-Cu_*x*_O encapsulation layer. So, it is likely that the amorphous encapsulation layer of h-Cu_*x*_O would transform into crystal CuO during electrochemical activation, which will cause the construction of Cu_2_O/CuO heterojunction in the h-Cu_*x*_O-EA sample.

The DFT calculations explored the catalytic process occurring at the electrodes further. The models of glucose absorbed on Cu_2_O, CuO, and Cu_2_O/CuO are presented in Fig. S26. The binding energy of glucose adsorption on Cu_2_O and CuO and Cu_2_O/CuO surface calculated is −0.254, −0.022, and −0.193 eV, respectively. The Cu_2_O has the smallest glucose adsorption energy. In contrast, the CuO has the largest among these 3 samples, which means that the glucose is harder to absorb onto the CuO surface but is easier to absorb onto Cu_2_O and Cu_2_O/CuO surfaces. Figure 4l shows the charge density map of glucose molecules absorbed on the Cu_2_O/CuO model surface, where the cyan indicates the charge depletion and yellow indicates charge accumulation. It is observed that the absorbed glucose molecular is surrounded by electron accumulation, indicating that the glucose plays an electron acceptor role. The corresponding electron received by glucose molecular is + 0.023*e* on Cu_2_O/CuO surfaces by Bader charge analysis. Besides the glucose adsorption process, the final product desorption procedure also affects the reaction speed. From the reaction pathway of glucose to gluconolactone (Fig. 4m), the glucose molecule is absorbed to the electrode surface firstly to form C_6_H_12_O_6_*, then the C–H bonds break spontaneously to get C_6_H_10_O_6_*. The desorption of C_6_H_10_O_6_* also affects the glucose oxidation reaction because C_6_H_10_O_6_* species hinder the glucose molecular adsorption. The desorption-free energy change of C_6_H_10_O_6_* on Cu_2_O, and CuO and Cu_2_O/CuO are 0.229, 0.321, and 0.064 eV, respectively, as shown in Fig. S27. So Cu_2_O/CuO has the fastest C_6_H_10_O_6_* desorption speed while CuO has the slowest. Generally speaking, the Cu_2_O/CuO has a relatively low glucose adsorption energy and the lowest C_6_H_10_O_6_* desorption energy, simultaneously contributing to outstanding glucose-sensing performance. It can be concluded that the Cu_2_O/CuO heterojunction structure accelerates the glucose oxidation reaction.

### Miniaturization for Glucose Detection Device

**Fig. 5:**
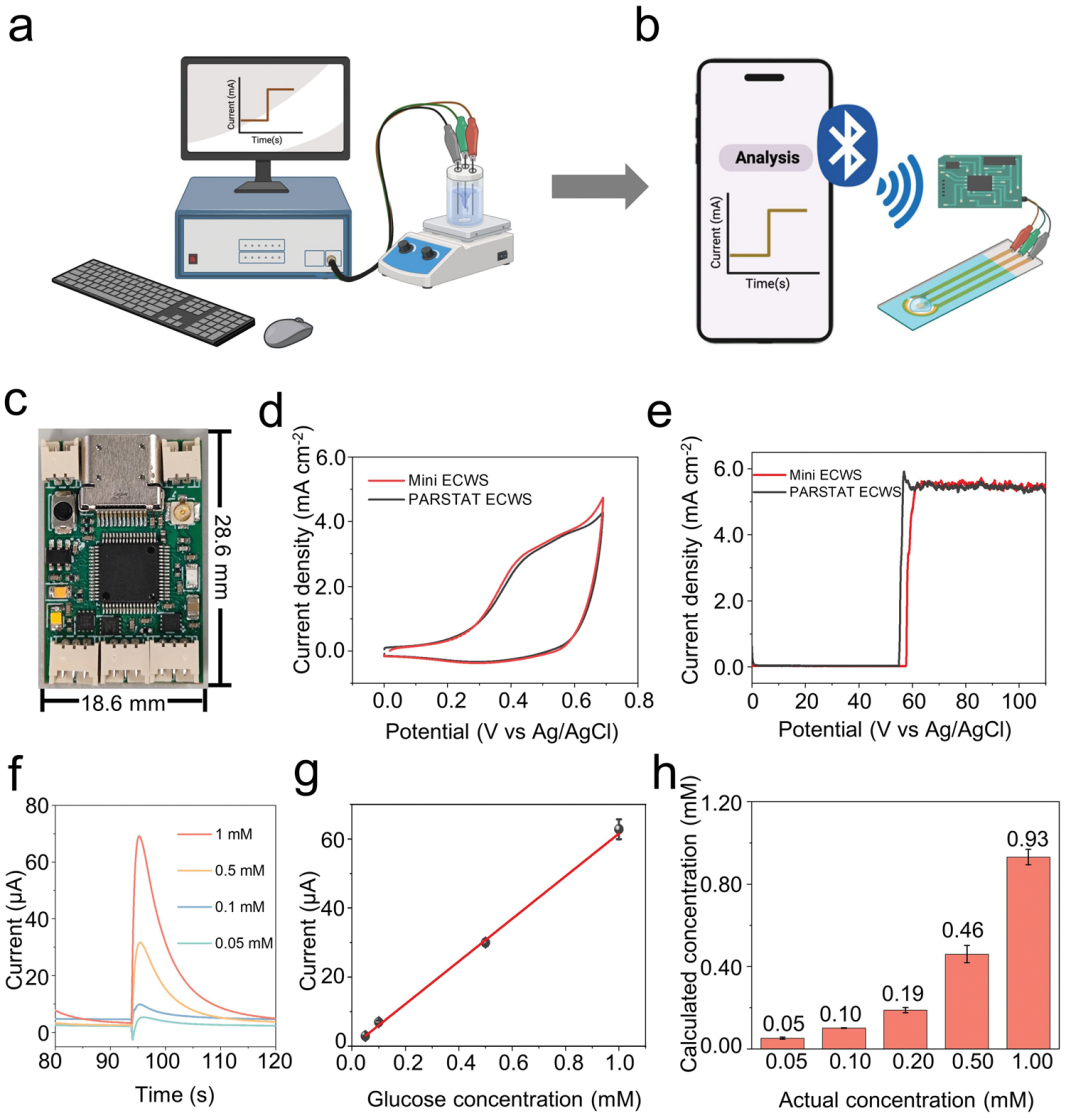
Miniaturization for glucose detection device. **a** Traditional glucose detection system. **b** Miniaturized glucose detection system. **c** Optical image of the mini electrochemical workstation. **d** CV comparison by mini and large electrochemical workstation in 0.1 M KOH electrolyte with 0.5 mM glucose. **e** Amperometric responses comparison by mini and large electrochemical workstation with adding 0.5 mM glucose. **f** Amperometric response to a series of glucose concentrations by a mini electrochemical workstation system. **g** Linear fitting curves of amperometric response with different glucose concentrations by miniaturized glucose detection system. **h** Relationship of calculated glucose concentration with actual glucose concentration in the artificial sweat by miniaturized glucose detection system

The traditional glucose detection system typically comprises a computer, an electrochemical workstation, and an electrolytic cell, which is very large and inconvenient (Fig. 5a). To solve this problem, a mini electrochemical workstation was established (Fig. 5b). The cumbersome three-electrode electrolytic cell system was replaced with a commercially available screen-printed electrode with pasted h-Cu_*x*_O-EA sample, 50 μL of 0.1 M KOH as electrolyte was then added to the electrode as shown in Fig. S28. Moreover, bluetooth can instantly read the data on the mobile phone. The photograph of the mini electrochemical workstation shows that its length and width are 28.6 and 18.6 mm, respectively (Fig. 5c). The design circuit diagram is illustrated in Fig. S29.

The CV curve of the mini electrochemical workstation is similar to the CV of the PARSTAT electrochemical workstation in 0.1 M KOH containing 0.5 mM glucose (Fig. 5d), indicating the reliability of the mini electrochemical workstation. Figure 5e demonstrates the amperometric responses of the h-Cu_*x*_O-EA to 0.5 mM glucose by the mini electrochemical workstation and PARSTAT electrochemical workstation. The results of these 2 electrochemical workstations are also close, further ensuring the reliability of the mini electrochemical workstation. Figure 5f shows the current response of h-Cu_*x*_O-EA on screen-printed electrode to different glucose concentrations by the mini electrochemical workstation system in which 5 μL corresponding glucose solution was dropped into 50 μL 0.1 M KOH, and Video S4 demonstrates this process. It is observed that the higher glucose concentration leads to a higher current response. The corresponding fitting curve is shown in Fig. 5g, which concludes that the amperometric response increases proportionally with the rise in glucose concentration, and the sensitivity is 61.67 μA mM^−1^. Five kinds of artificial sweat with different glucose concentrations were tested, and the calculated glucose concentration was close to the actual glucose concentration.

## Conclusions

In this work, we report a novel laser-induced transfer technique for crafting portable glucose sensors by repurposing copper from electronic waste. Our method employs a fully automated laser-induced process to synthesize continuous h-Cu_*x*_O nano-skeletons tailored for glucose sensing. This approach offers swift, environmentally friendly, air-compatible, and continuous fabrication, suitable for various Cu-containing substrates. Utilizing this method, we produce h-Cu_*x*_O nano-skeletons from discarded PCBs. These nano-structures feature an inner layer primarily composed of Cu_2_O with a lower oxygen content, juxtaposed with an outer layer rich in amorphous Cu_*x*_O with a higher oxygen content. When applied in glucose detection, these heterogeneous nano-skeletons undergo a structural transformation into rigid Cu_2_O@CuO nano-skeletons upon electrochemical activation. This transformation results in exceptional performance, with a sensitivity of 9.893 mA mM^−1^ cm^−2^ and a low detection limit of 0.34 μM. DFT analysis elucidates the effectiveness of the heterogeneous structure in facilitating gluconolactone desorption. Furthermore, we have miniaturized this glucose detection device to enhance its scalability and portability for convenient integration into people’s daily lives.

Looking to the future, this method provides a new approach to the high-value utilization of discarded PCBs. By precisely controlling the laser-treated area, we can prepare high-performance glucose-sensing catalysts while simultaneously recovering copper. This method exhibits strong potential for scalable production by integrating with existing industrial workflows, contributing to resource efficiency and sustainability. However, industrial implementation necessitates further investigation into the long-term stability and reliability of the catalysts and optimization of the integrated process for commercial viability. Additionally, exploring the adaptability of this method for fabricating other sensor types is crucial to unlocking the full application potential of waste PCBs. Through collaborative efforts between researchers and industry, we believe this waste PCB-derived glucose-sensing catalyst holds promise for near-future practical applications, offering a cost-effective, efficient, and sustainable solution for diabetic health monitoring.

## Supplementary Information

Below is the link to the electronic supplementary material.

Supplementary file1 (PDF 2141 KB)

Supplementary file2 (MP4 5983 KB)

Supplementary file3 (MP4 840 KB)

Supplementary file4 (MP4 663 KB)

Supplementary file5 (MP4 4611 KB)
