# Supplementary material for: Heterogeneous CuxO Nano-Skeletons from Waste Electronics for Enhanced Glucose Detection
Source: Nanomicro Lett. 2024 Jul 18;16:249. doi: 10.1007/s40820-024-01467-5 (PMC11258110; doi:10.1007/s40820-024-01467-5)
Supplement: Supplementary file 1 — Supplementary file1 (PDF 2141 KB) [file 40820_2024_1467_MOESM1_ESM.pdf]

Supporting Information for

Heterogeneous Cu_xO Nano-Skeletons from Waste Electronics for Enhanced Glucose Detection

Yexin Pan[1, #], Ruohan Yu[1, 2, #], Yalong Jiang[3], Haosong Zhong[1], Qiaoyaxiao Yuan[1], Connie Kong Wai Lee[1], Rongliang Yang[1], Siyu Chen[1], Yi Chen[1], Wing Yan Poon[1], and Mitch Guijun Li[1,*]

[1]Center on Smart Manufacturing, Division of Integrative Systems and Design, The Hong Kong University of Science and Technology, Clear Water Bay, Kowloon, Hong Kong SAR 999077, P. R. China

[2] The Sanya Science and Education Innovation Park of Wuhan University of Technology, Sanya 572000, P. R. China

[3] State Key Laboratory of New Textile Materials and Advanced Processing Technologies, Wuhan Textile University, Wuhan 430200, P. R. China

[#]Yexin Pan and Ruohan Yu contributed equally to this work.

*Corresponding author. E-mail: mitchli@ust.hk (Mitch Guijun Li)

Supplementary Figures

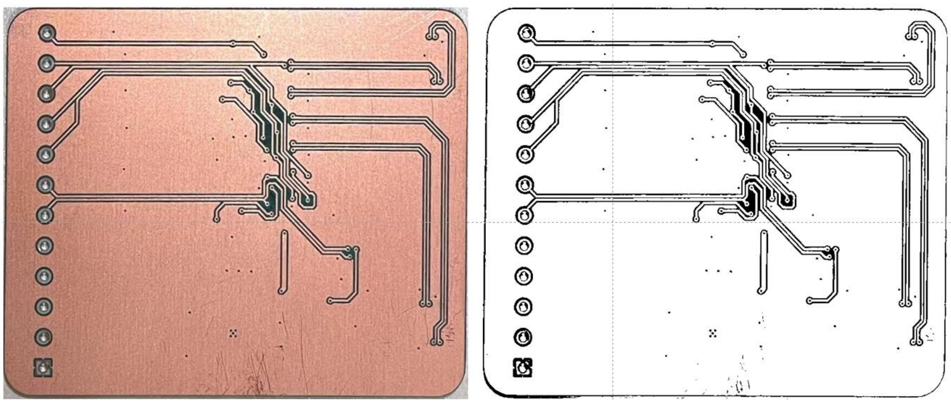

Fig. S1 The optical and corresponding grayscale images of the PCB

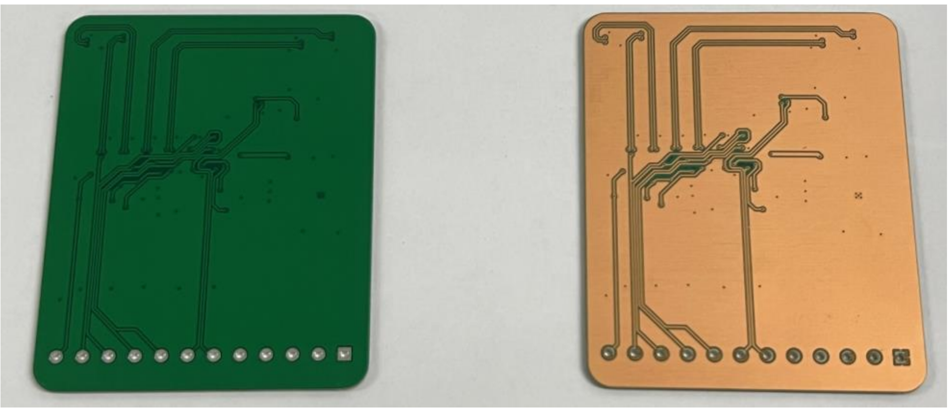

Fig. S2 The optical photo of PCB before and after varnish removal

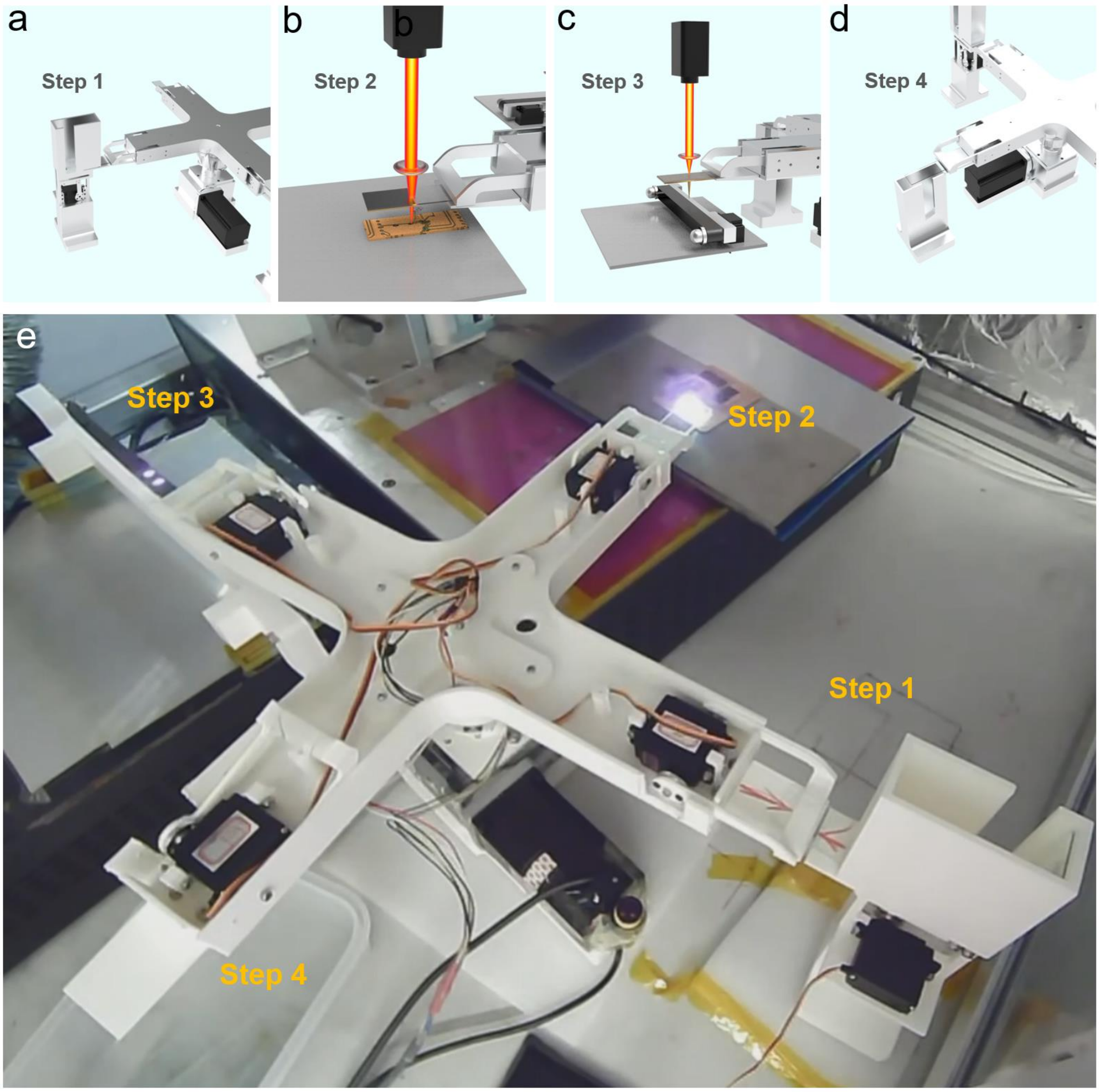

Fig. S3 The procedures and optical image of the fully automatic fabrication system

Note: We designed and fabricated this system. The system comprises four main components: a spin robot equipped with four arms positioned at 90-degree intervals for holding glass sheets, a glass sheet injector supplying glass sheets to the robot arm, a carbon cloth conveyor for transporting carbon cloth, and a laser system. Fabricating the working electrode involves four steps in this automated system. First, a robot arm retrieves a glass sheet from the injector (Fig. S3a, Step 1). Then, the arm rotates 90 degrees, positioning the glass sheet over the PCB for the LIBT process, which recycles copper from waste PCB onto the glass sheet (Fig. S3b, Step 2). Next, the arm moves to the carbon cloth conveyor, initiating a LIFT process to transfer copper from the glass sheet to the carbon cloth (Fig. S3c, Step 3). Once completed, the carbon cloth shifts 2 cm to expose raw material for laser treatment in the subsequent LIFT process. Finally, used glass sheets are removed and stored (Fig. S3d, Step 4). With this automated system, electrodes can be continuously produced once laser fabricating parameters are established, and the optical image of the fully automatic fabrication system is shown as Fig. S3e. Copper transferred onto the carbon cloth serves directly as the working electrode for glucose detection.

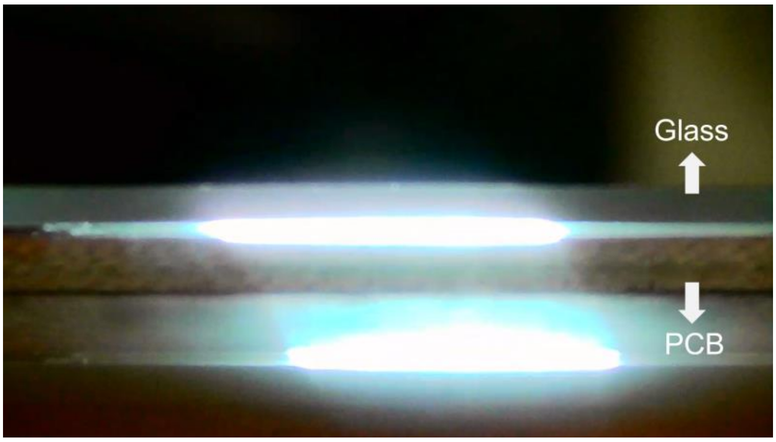

Fig. S4 Record image of PCB LIBT process

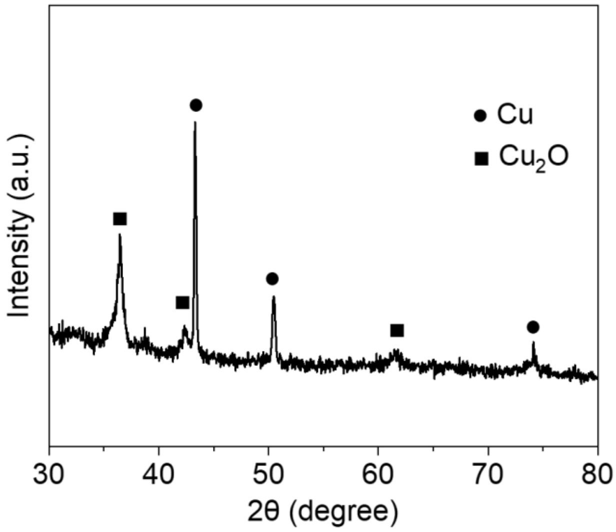

Fig. S5 XRD pattern of PCB Cu LIBT to glass sample

Note: The XRD result shows that the copper underwent partially oxidation and formed the mixture of Cu_2O and Cu after LIBT process.

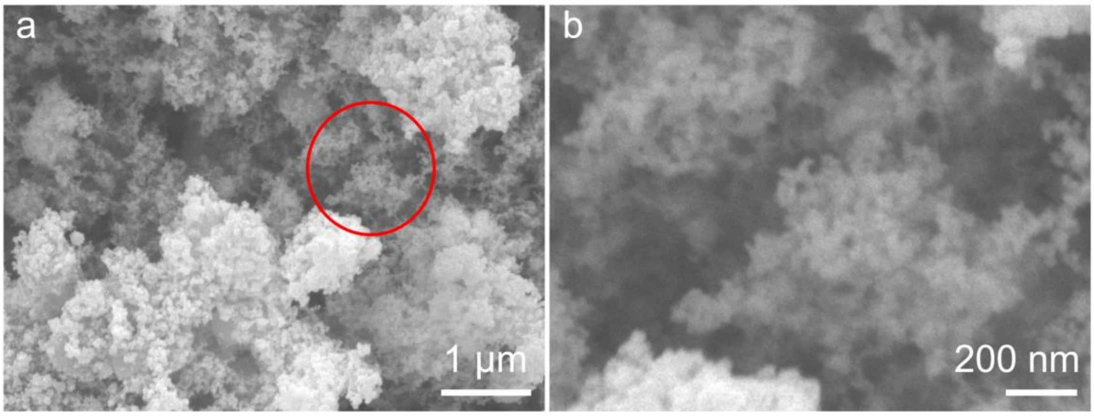

Fig. S6 a SEM image of PCB Cu LIBT to glass sample. **b** magnified image from the labeled region in **a**

Note: The SEM image shows that the bulk copper became bridged nanoparticles after the LIBT process.

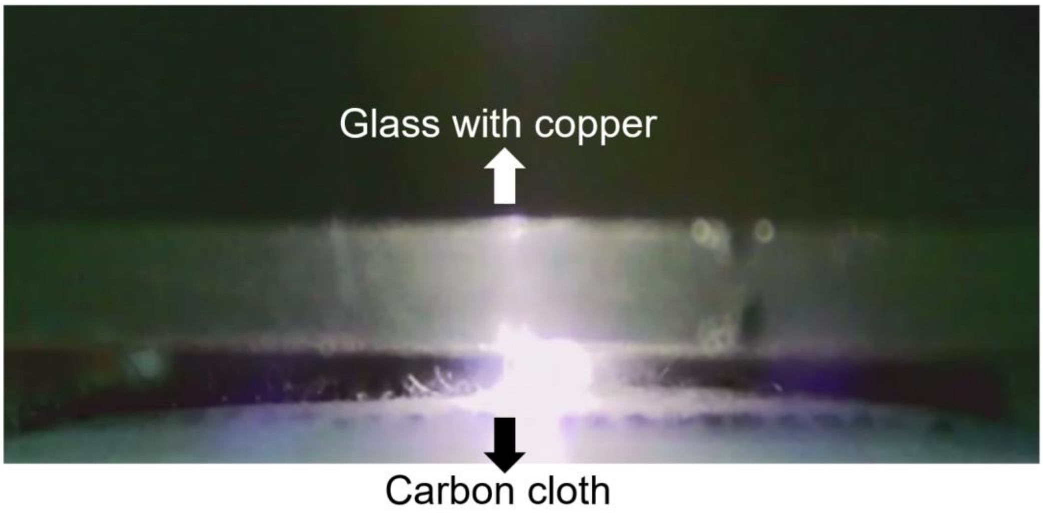

Fig. S7 Record image of PCB LIFT process

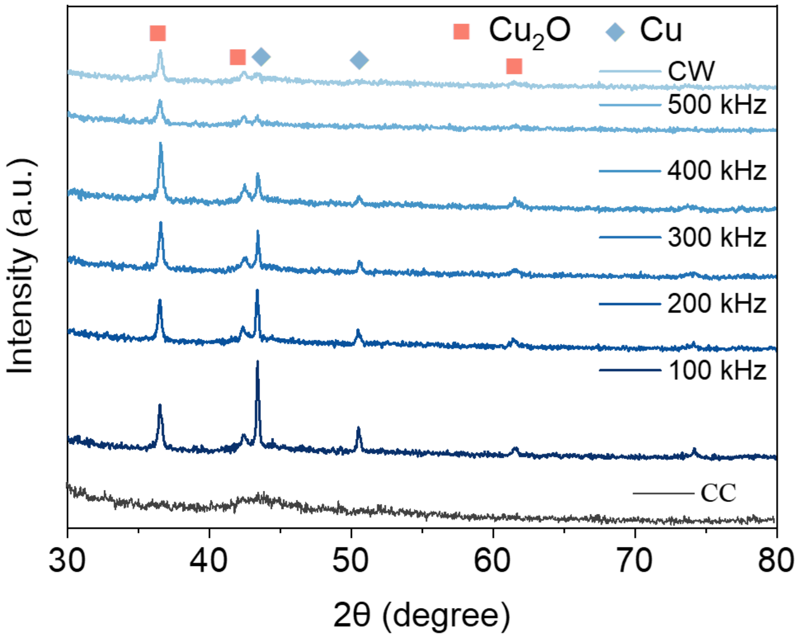

Fig. S8 XRD of samples by different frequency (CC represents the pure carbon cloth treated by laser with CW mode)

Note: The XRD result shows that the samples gotten under relatively low laser frequency are the mixture of Cu_2O and Cu. And the peaks intensity corresponding to Cu decreases with the increasing of frequency until the peak of Cu almost disappears when the laser beam is working in continuous-wave (CW) mode, indicating the sample under CW moder is Cu_2O.

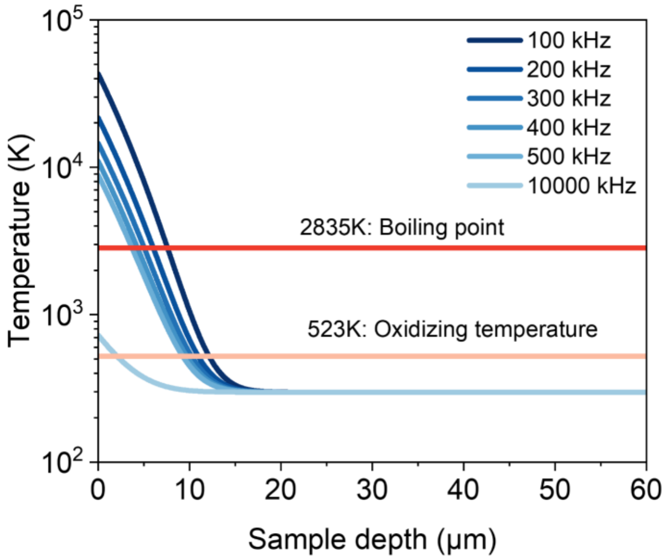

Fig. S9 Calculated rising temperature vs. donor depth at the center of the laser beam (*r* = 0).

Note: To further investigate the oxidation kinetics of the forward-transferred copper particles under different laser frequencies, we developed a simplified model for computing the temperature distributions within the donor layer when irradiated by a single laser pulse. The calculation principle is referenced from the classical laser radiation theory [S1] and our previous report [S2]. The donor material experiences a transient increase in temperature to an ultra-high level when absorbing laser energy. The rising temperature ΔT can be calculated based on a function about the radial distance from the donor's surface (*r*), the depth of the donor layer (*z*), and processing time (*t*):

$$\Delta T(r,z,t) = \frac{I_{max}(1-R)\sqrt{K}}{\sqrt{\pi}\gamma}\int_0^{\tau}\frac{p(\tau-t)}{\sqrt{t}[1+\frac{8Kt}{W^2}]}exp\left[-\frac{z^2}{4Kt}-\frac{r^2}{4Kt+0.5W^2}\right]dt \qquad \text{(S1)}$$

The temperature increase as a function donor depth at the center of the laser beam (r = 0) was calculated using Eq. (S1), which takes into account the pulse intensity (I_{max}), Fresnel energy reflectivity (*R*), copper's thermal diffusivity (*K*), copper's thermal conductivity (γ), laser beam's mode field radius (*W*), and pulse width (τ). For simplicity, a square-shaped pulse was assumed, determined by the temporal function *p*(t).

As demonstrated in the curves of calculated rising temperature vs. donor depth (Fig. S8), the laser pulses with frequencies from 100 to 500 kHz contribute to the ultra-high transient temperatures (~8,000 to 40,000 K) in the near-surface of the donor. These calculated theoretical temperatures are far higher than the boiling point of copper. Vaporization and even ionization will occur in the irradiated copper particles. Apparent plasma generation can be observed during the LIFT process. The rapid generation and expansion of copper vapor/plasma will result in the formation of laser-supported detonation waves (LSD), promoting the high-speed transfer of copper-based particles [S3, S4]. The laser pulse with a lower laser frequency will lead to a higher surface temperature of the donor, contributing to a more intense micro-detonation and a more rapid transfer process.

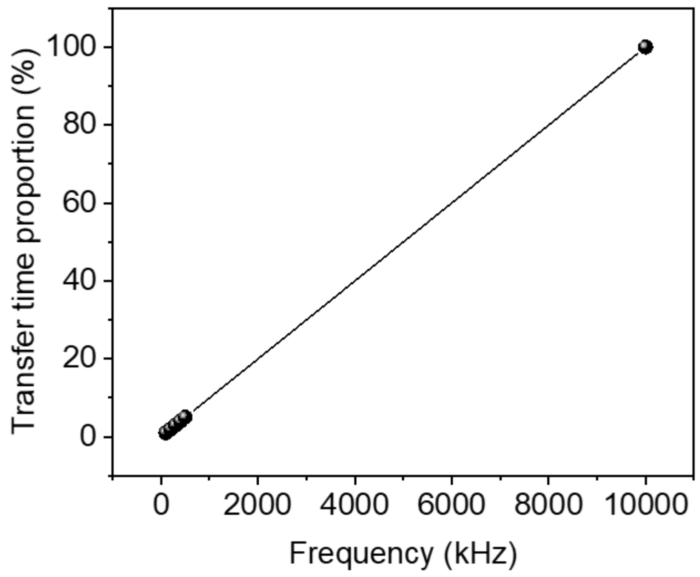

Fig. S10 The influence of laser frequency to transfer time proportion

Note: The proportion of actual laser transfer time (p (%)) under a single laser pulse irradiation is calculated via **Eq. (S2)**:

$$p\ (\%) = f_L \tau_L \times 100\% \qquad \text{(S2)}$$

Figure S10 shows the transfer time positively correlates with the laser frequency under the irradiation of a single laser pulse

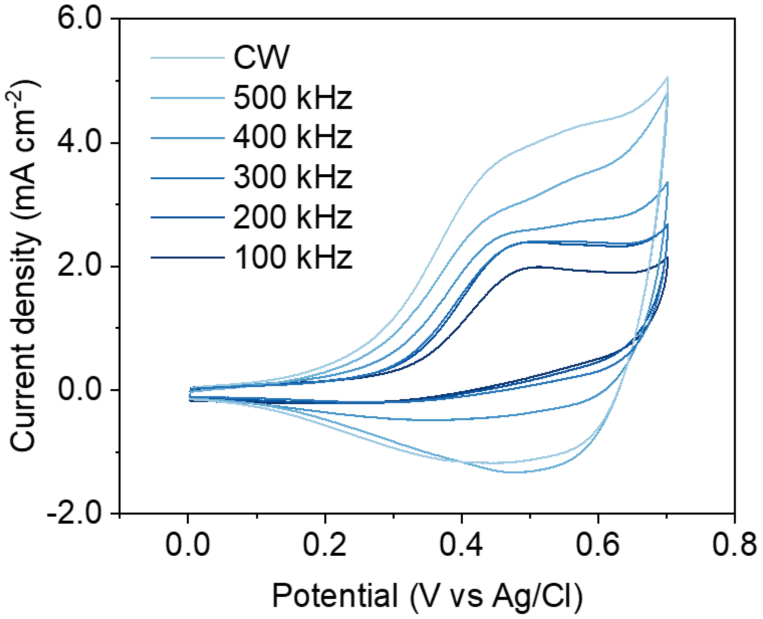

Fig. S11 CV of samples by different laser frequency with 0.5 M glucose at 50 mV/s

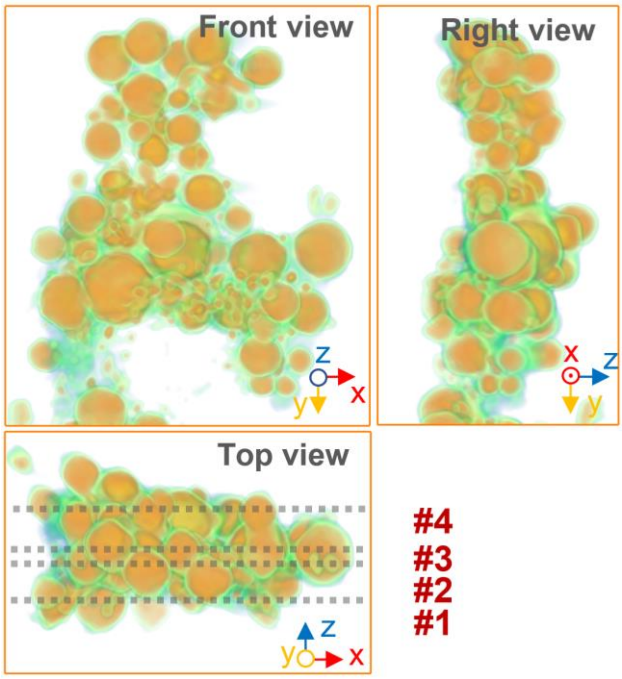

Fig. S12 Three-view drawing of h-Cu$_x$O 3-dimensional tomography reconstructed mode

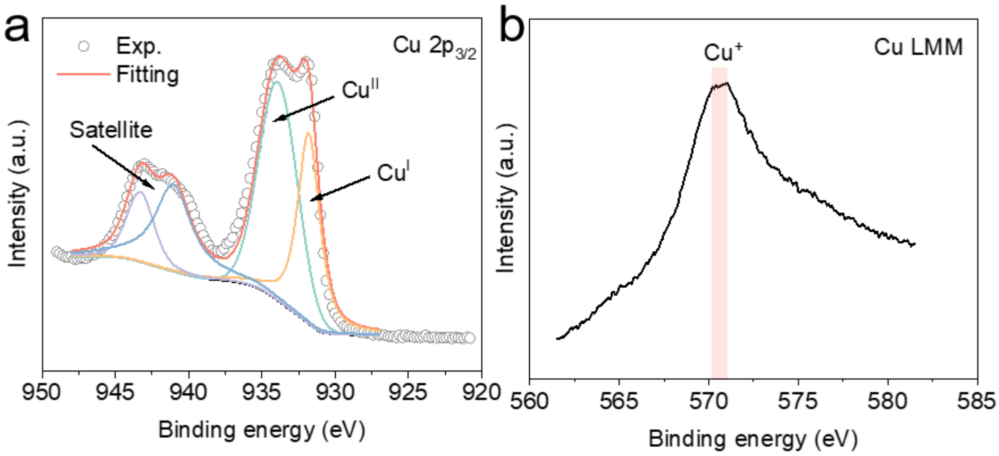

Fig. S13 a Cu 2p XPS. **b** Cu LMM of h-Cu$_x$O sample

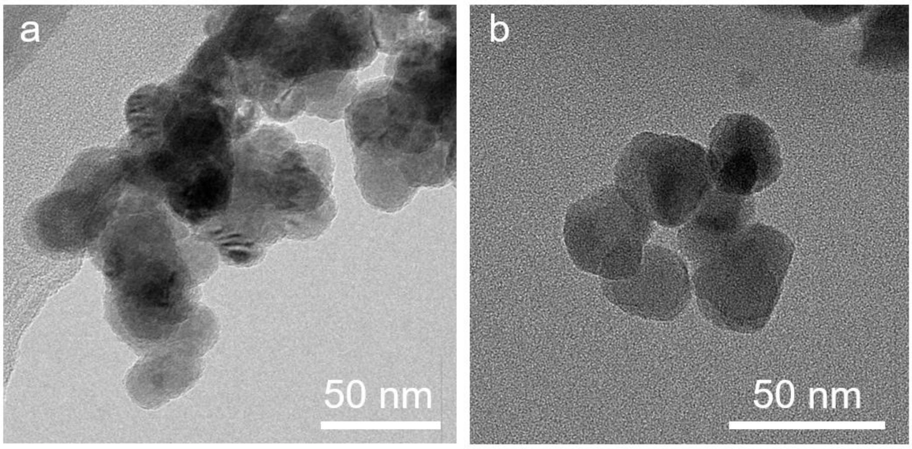

Fig. S14 TEM image of **a** commercial Cu_2O and **b** CuO nanoparticles

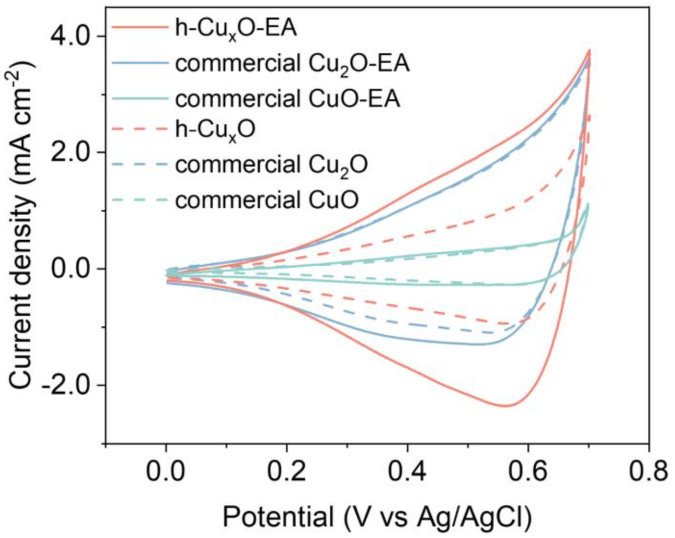

Fig. S15 CV curves of the h-Cu$_x$O, commercial Cu$_2$O, and CuO electrodes before and after CA activation

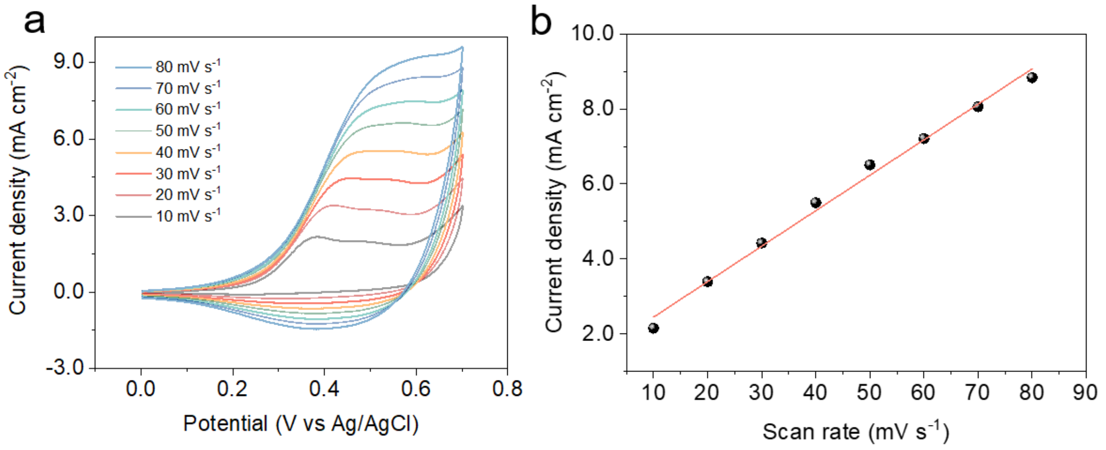

Fig. S16 a CV curves of the h-Cu$_x$O-EA electrode with 1 mM glucose at series of scan rates. **b** fitting curve of anodic peak current density versus scan rates

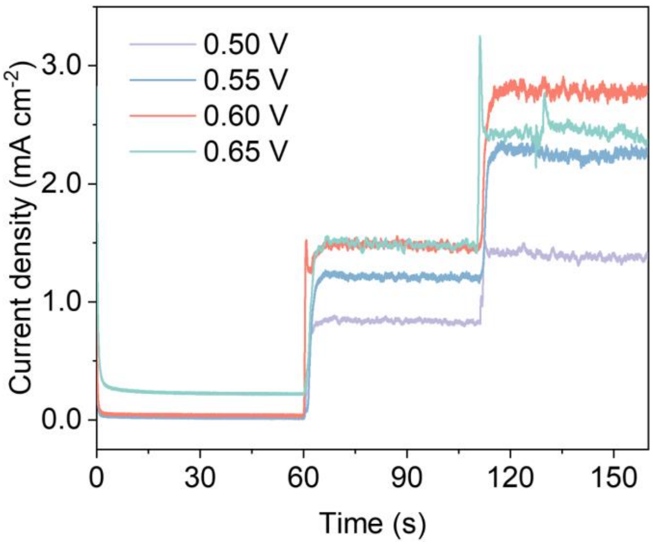

Fig. S17 Amperometric responses of h-Cu$_x$O-EA to 0.1 M glucose at various potentials

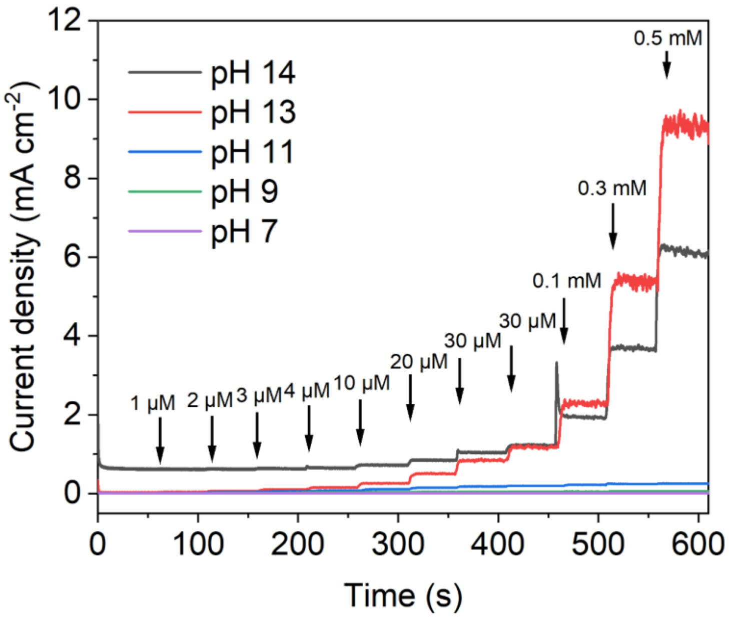

Fig. S18 Amperometric responses of h-Cu$_x$O-EA to various glucose at various pH electrolyte

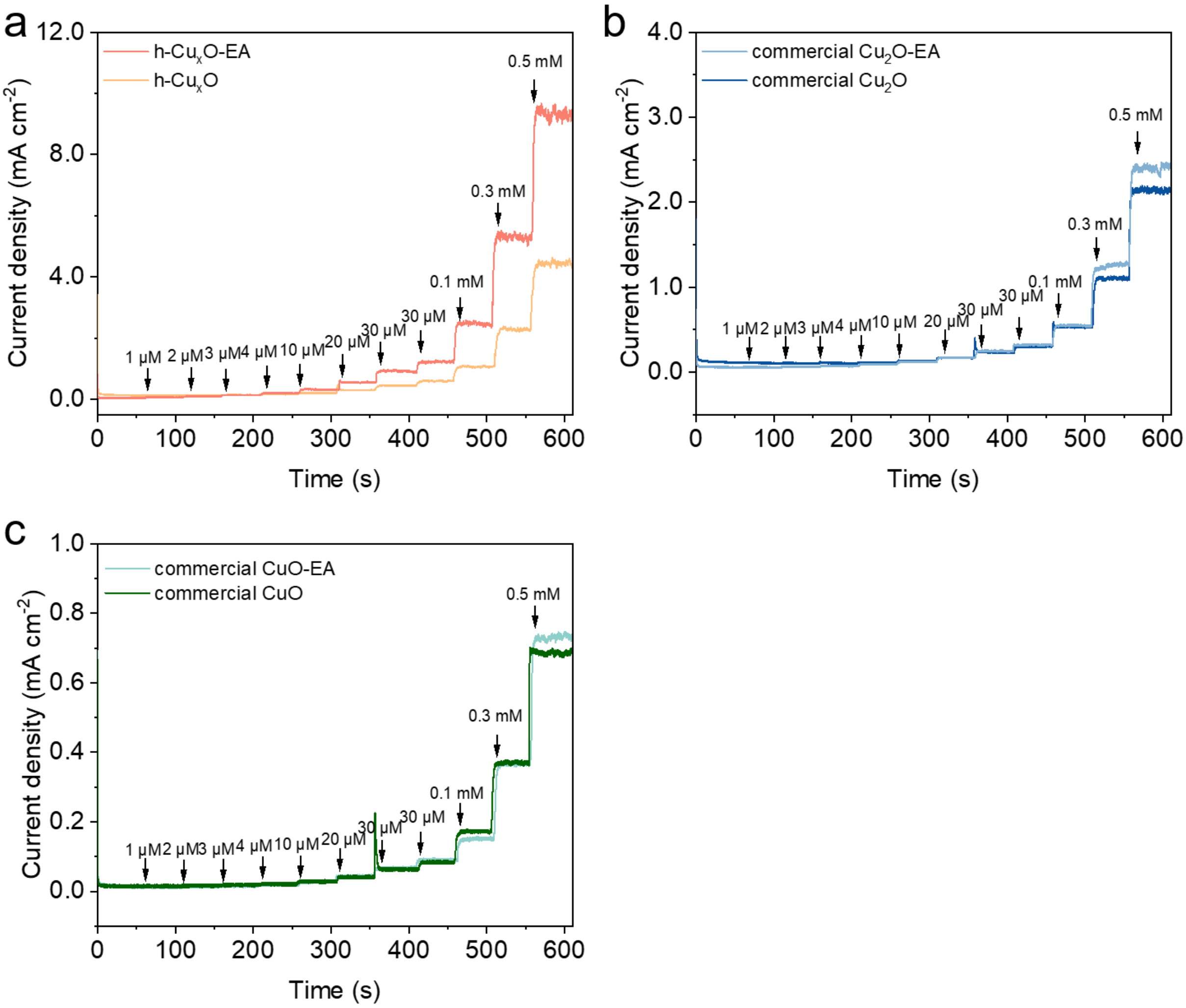

Fig. S19 Amperometric responses of **a** h-Cu$_x$O-EA and h-Cu$_x$O, **b** commercial Cu_2O-EA and Cu_2O, **c** commercial CuO-EA and CuO to various concentration of glucose

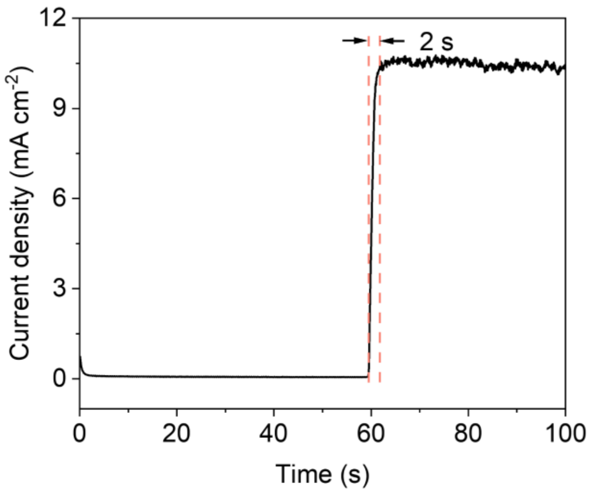

Fig. S20 Amperometric responses of h-Cu$_x$O-EA to 1 M glucose in 0.1 M KOH at 0.6 V vs Ag/AgCl

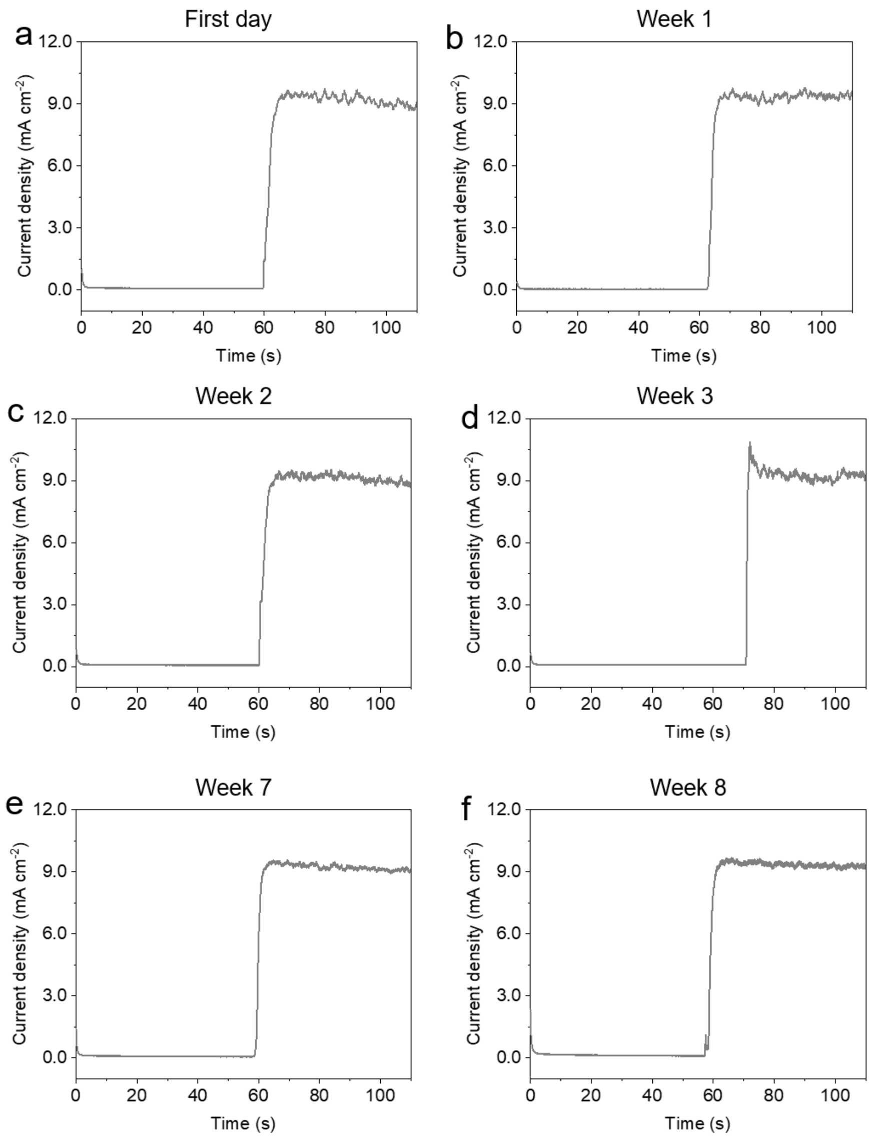

Fig. S21 Amperometric responses of h-Cu$_x$O-EA to 1 mM glucose with time: **a** the first day. **b** week 1. **c** week 2. **d** week 3. **e** week 7. **f** week 8

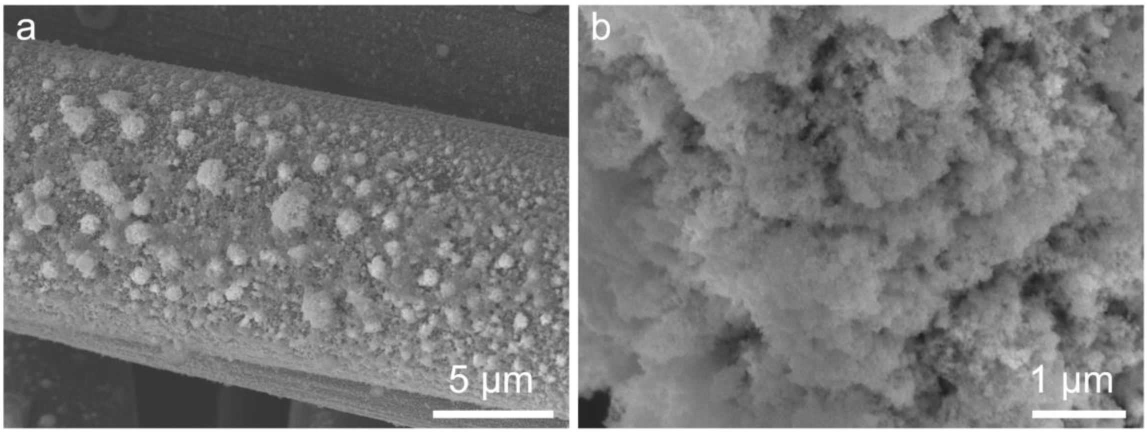

Fig. S22 SEM image of h-Cu$_x$O-EA

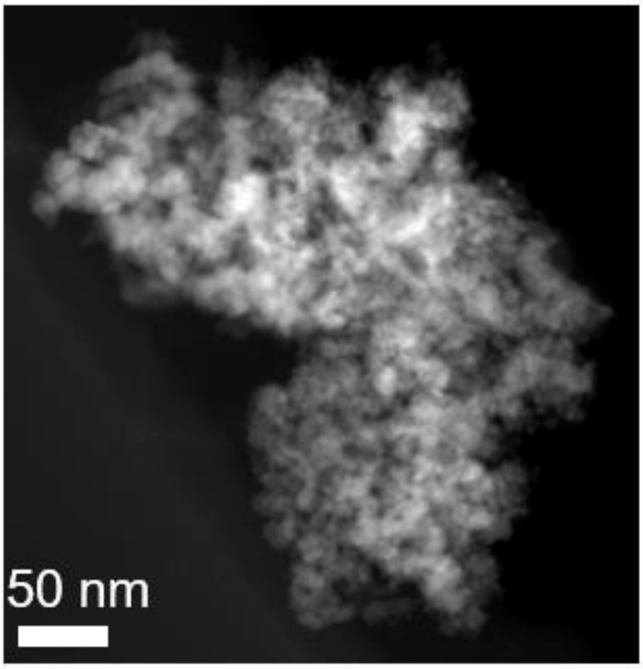

Fig. S23 TEM of h-Cu$_x$O-EA

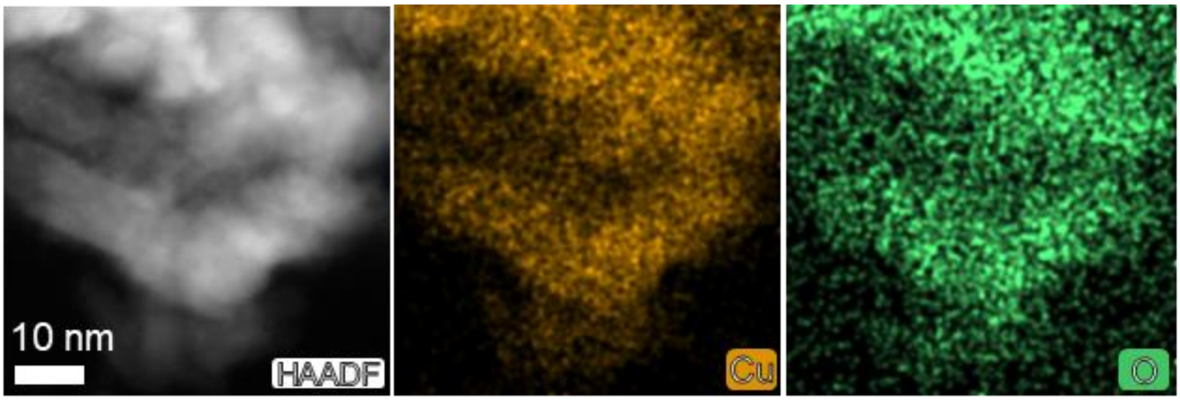

Fig. S24 HADDF image and corresponding EDX mapping of h-Cu$_x$O-EA

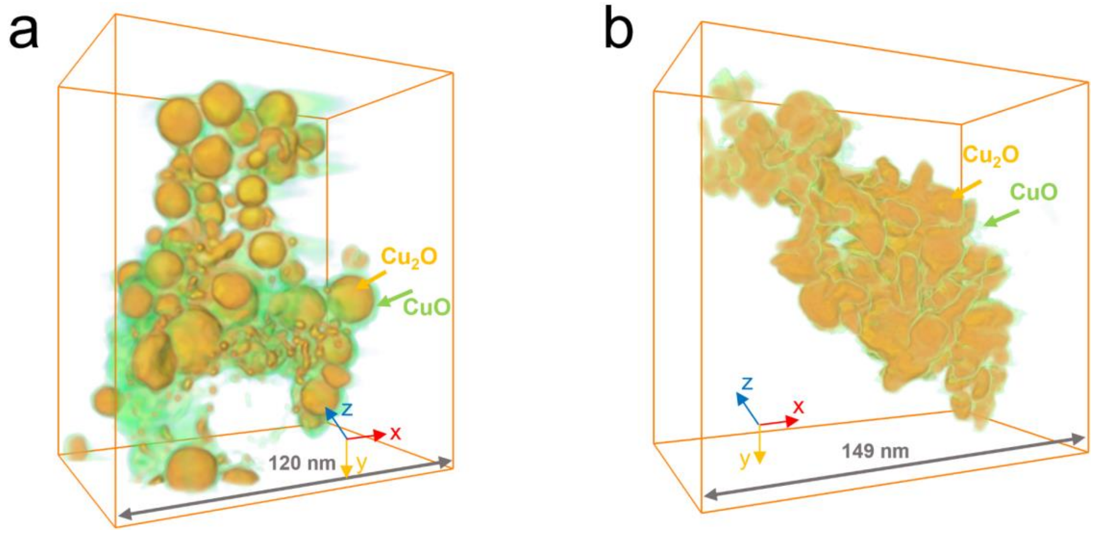

Fig. S25 Reconstructed model of (**a**) h-Cu$_x$O and (**b**) h-Cu$_x$O-EA samples

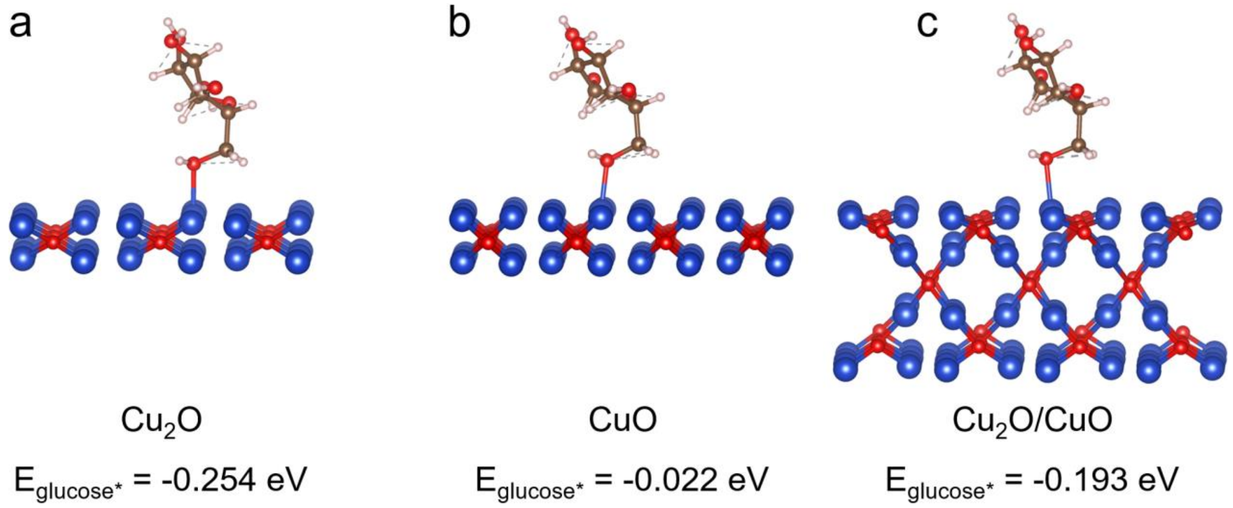

Fig. S26 The DFT calculation models of a, Cu_2O; b, CuO and c, Cu_2O/CuO

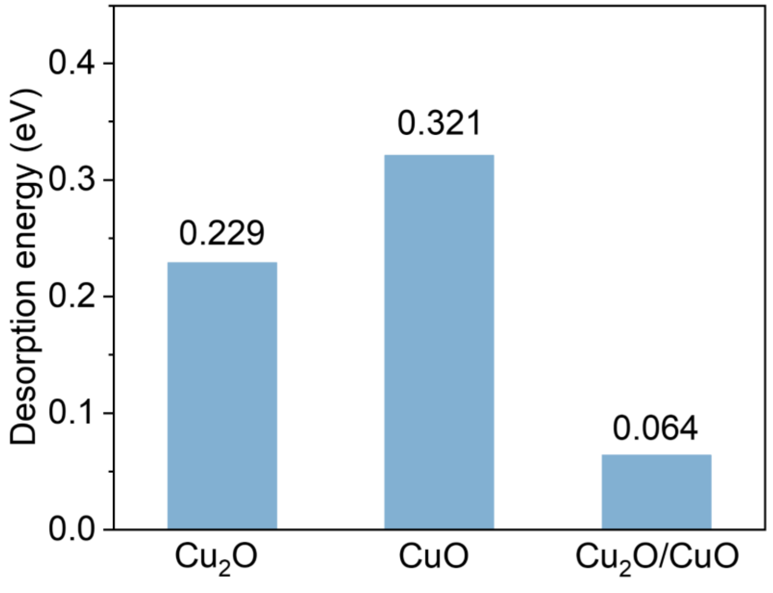

Fig. S27 The desorption free energy change on $C_6H_{10}O_6$* for Cu_2O, and CuO and Cu_2O/CuO

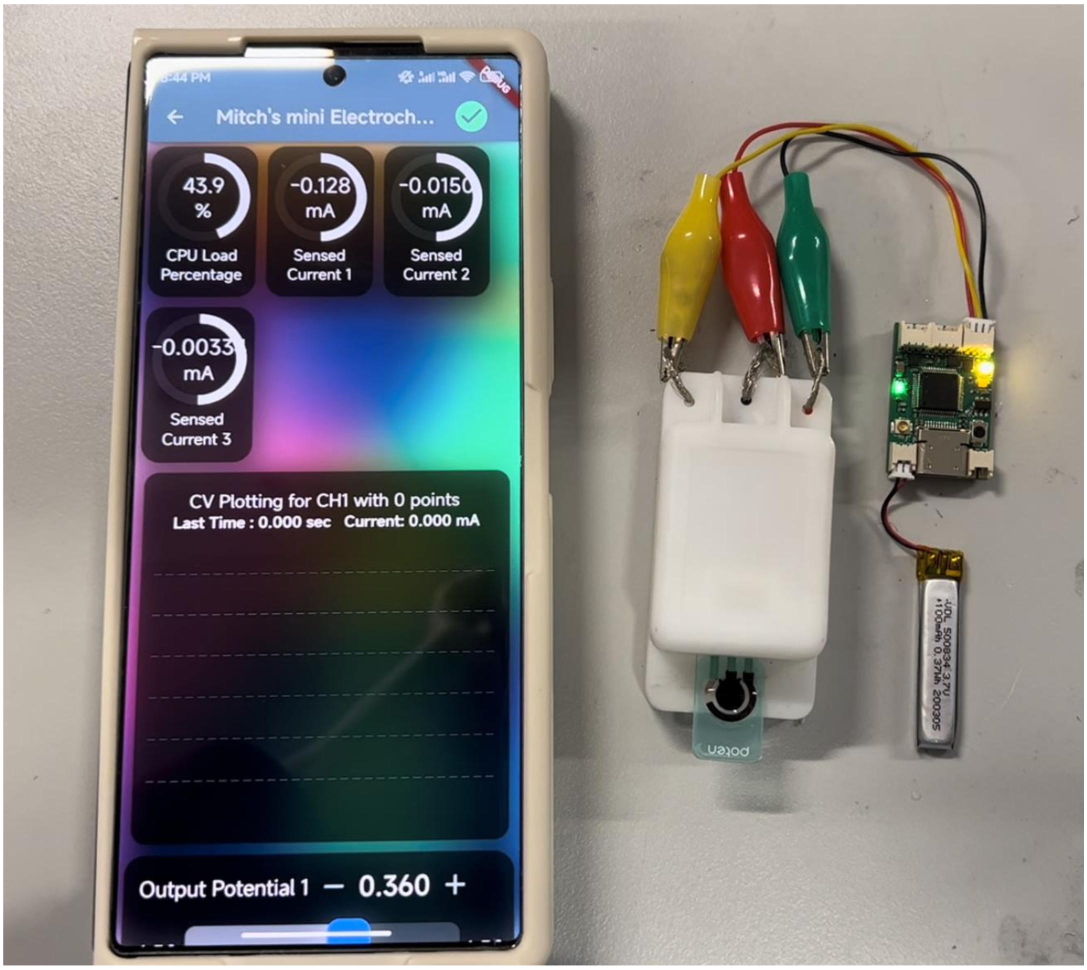

Fig. 28 The photo of Miniaturized detection device

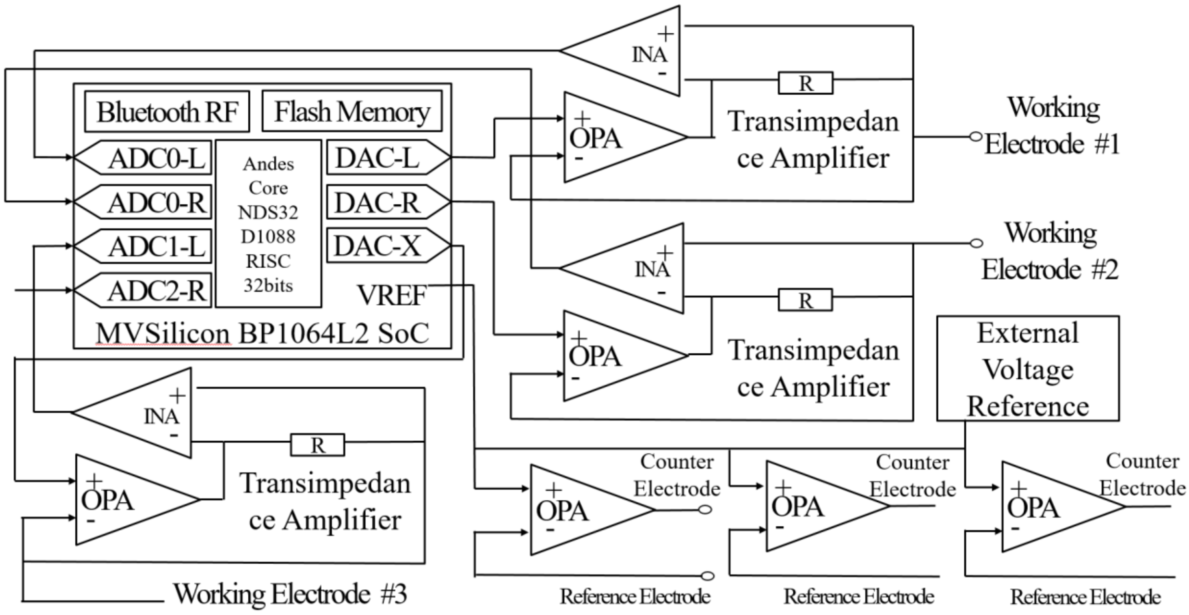

Fig. S29 The circuit design of mini electrochemical workstation

Table S1 The mass reduction of the PCB *vs*. the corresponding mass increase of the glass sheet

Mass reduction of PCB (mg)	Mass increase of glass sheet (mg)	Recycling efficiency (%)
1.1	0.91	82.73
1.23	1.1	89.43
1.38	1.04	75.36

Table S2 Measured laser output power, calculated light intensity, and calculated pulse energy fluence *vs*. set laser operating power and laser frequency

Set			Measured	Calculated	
Operating power (W)	Pulse frequency (kHz)	Pulse width (ns)	Output power (W)	Pulse energy fluence (J cm^{-2})	Light intensity (10^7 W cm^{-2})
18	100	100	6.44	13.12	13.12
18	200	100	6.44	6.56	6.56
18	300	100	6.44	4.37	4.37
18	400	100	6.44	3.28	3.28
18	500	100	6.44	2.62	2.62
18	10000	100	6.44	0.13	0.13

Note: Calculation in LIFT process

The calculation principle is referenced from the classical laser radiation theory and our previous report [S1, S2]. The operation software sets the operating powers P_0, pulse frequency f_L, and pulse width τ_L. The laser power meter was used to test the laser output power (*P*). **Equations (3) and (4)** can be used to calculate the light intensity *I* and pulse energy fluence *F*, respectively:

$$I = F/\tau_L = 4P/\pi f_L \tau_L D_L^2 \quad \text{(S3)}$$

$$F = 4P/\pi f_L D_L^2 \quad \text{(S4)}$$

As shown in Table S2, with the fixed operating power and pulse width, the laser output laser pulses deliver a constant average output power of 6.44 W. Therefore, the pulse energy fluences decrease from 13.12 to 2.62 J cm^{-2} when the pulse frequencies increase from 100 to 500 kHz. It should be noted that in a near-continuous laser mode (at 100 ns pulse width and ~10000 pulse frequency), the energy fluence corresponding to every single pulse is low (0.13 J cm^{-2}).

Table S3 Comparison of the glucose detection performance of various electrodes

Electrodes	Preparation method	Approximate Preparation time	Sensitivity (μA mM^{-1} cm^{-2})	LOD (μM)	Refs.
CuO nanoflakes	One-pot method	6 h	4870	0.5	[S5]
$NiCo_2O_4$@Ppy	Hydrothermal and calcination	40 h	3059	0.22	[S6]
CuO/Cu_2O	Hydrothermal method	14 h	1541	0.57	[S7]
CuO nanoflowers	Colloidal and combustion based techniques	3 h	2062	0.25	[S8]
Pt_1/Cu@CuO	Oil bath	56 h	852.163	3.6	[S9]
Cu/Cu_2O@C	Chemical coprecipitation and calcination	3 days	621.12	0.31	[S10]

CuO/Cu_2S	Chemical oxidation and calcination	20 h	2680	0.95	[S11]
CuO/$Ni(OH)_2$/CC	Hydrothermal method	8 h	598.6	0.31	[S12]
CuO/Cu_2O	Chemical coprecipitation and calcination	35 h	2560.8	0.55	[S13]
Co-Bi NPs	Laser ablation	4 h	2326	4	[S14]
Ni-LCNFs	Electrospinning and laser carbonization	13 h	2092	0.3	[S15]
NiCo-BTC/CC	Hydrothermal method	15 h	2701.29	0.09	[S16]
NiO/Co_3O_4/C	Hydrothermal and annealing method	40 h	2820	0.045	[S17]
NiO/Ni foam	Hydrothermal method	18 h	2739.5	0.75	[S18]
ZnO/MXene/GO_x	Thermal evaporation and etching method	52 h	29	17	[S19]
BPCNFs/GO_x	pyrolyzation	26 h	123.28	0.023	[S20]
**h-Cu_xO-EA**	**Laser induced transfer method**	**< 1 min**	**9893**	**0.34**	**This work**

Supplementary References

[S1] S. T. Hendow, S. A. Shakir. Structuring materials with nanosecond laser pulses. Opt. express. **18**(10), 10188-10199 (2010). https://doi.org/10.1364/OE.18.010188

[S2] Y. Huang, R. Yang, H. Zhong, C. K. W. Lee, Y. Pan et al., High-throughput automatic laser printing strategy toward cost-effective portable integrated urea tele-monitoring system. Small Methods 2301184 (2023). https://doi.org/10.1002/smtd.202301184

[S3] M. R. Maina, Y. Okamoto, K. Hamada, A. Okada, S.-i. Nakashiba et al., Effects of superposition of 532 nm and 1064 nm wavelengths in copper micro-welding by pulsed Nd: YAG laser. J. Mater. Process Technol. **299**, 117388 (2022). https://doi.org/10.1016/j.jmatprotec.2021.117388

[S4] W. Maher, R. Hall, R. Johnson. Experimental study of igition and propagation of laser-supported detonation waves. J. Appl. Phys. **45**(5), 2138-2145 (1974). https://doi.org/10.1063/1.1663559

[S5] S. Wang, H. Yang, X. Yi, H. M. K. Sari, X. Zhang et al., Significant influence of controllable surface oxygen vacancies of cuo for enhancing sensitivity of glucose detection. Appl. Surf. Sci. **574**, 151649 (2022). https://doi.org/10.1016/j.apsusc.2021.151649

[S6] X. Duan, K. Liu, Y. Xu, M. Yuan, T. Gao et al., Nonenzymatic electrochemical glucose biosensor constructed by $NiCo_2O_4$@Ppy nanowires on nickel foam substrate. Sens. Actuators B Chem. **292**, 121-128 (2019). https://doi.org/10.1016/j.snb.2019.04.107

[S7] J. Lv, C. Kong, Y. Xu, Z. Yang, X. Zhang et al., Facile synthesis of novel CuO/Cu_2O nanosheets on copper foil for high sensitive nonenzymatic glucose biosensor. Sens. Actuators B Chem. **248**, 630-638 (2017). https://doi.org/10.1016/j.snb.2017.04.052

[S8] A. Ashok, A. Kumar, F. Tarlochan. Highly efficient nonenzymatic glucose sensors based on cuo nanoparticles. Appl. Surf. Sci. **481**, 712-722 (2019). https://doi.org/10.1016/j.apsusc.2019.03.157

[S9] Y. Zhao, Y. Jiang, Y. Mo, Y. Zhai, J. Liu et al., Boosting electrochemical catalysis and nonenzymatic sensing toward glucose by single-atom Pt supported on Cu@CuO core-shell nanowires. Small **19**(18), 2207240 (2023). https://doi.org/10.1002/smll.202207240

[S10] X. Zhuang, C. Han, J. Zhang, Z. Sang, W. Meng. Cu/Cu_2O heterojunctions in carbon framework for highly sensitive detection of glucose. J. Electroanal. Chem. **882**, 115040 (2021). https://doi.org/10.1016/j.jelechem.2021.115040

[S11] L. Thi, T. H. Ho, T. V. Vu, D. L. Nguyen, M. X. Tran et al., P-incorporated CuO/Cu_2S heteronanorods as efficient electrocatalysts for the glucose oxidation reaction toward highly sensitive and selective glucose sensing. Phys. Chem. Chem. Phys. **26**(1), 249-260 (2024). https://doi.org/10.1039/D3CP04095J

[S12] S. Sun, N. Shi, X. Liao, B. Zhang, G. Yin et al., Facile synthesis of CuO/$Ni(OH)_2$ on carbon cloth for non-enzymatic glucose sensing. Appl. Surf. Sci. **529**, 147067 (2020). https://doi.org/10.1016/j.apsusc.2020.147067

[S13] H. Chen, L. Shao, J. Ma, W. He, B. Zhang et al., Hierarchical hollow CuO/Cu_2O and Cu_2O/Cu/C derived from metal–organic framework for non-enzymatic oxidation toward glucose. J. Mol. Liq. **375**, 121317 (2023). https://doi.org/10.1016/j.molliq.2023.121317

[S14] W. Zheng, Y. Li, L. Y. S. Lee. Bismuth and metal-doped bismuth nanoparticles produced by laser ablation for electrochemical glucose sensing. Sens. Actuators B Chem. **357**, 131334 (2022). https://doi.org/10.1016/j.snb.2021.131334

[S15] M. Simsek, K. Hoecherl, M. Schlosser, A. J. Baeumner, N. Wongkaew. Printable 3d carbon nanofiber networks with embedded metal nanocatalysts. ACS Appl. Mater. Interfaces **12**(35), 39533-39540 (2020). https://doi.org/10.1021/acsami.0c08926

[S16] X. Zha, W. Yang, L. Shi, Y. Li, Q. Zeng et al., Morphology control strategy of bimetallic mof nanosheets for upgrading the sensitivity of noninvasive glucose detection. ACS Appl. Mater. Interfaces **14**(33), 37843-37852 (2022). https://doi.org/10.1021/acsami.2c10760

[S17] A. Vignesh, P. Vajeeston, M. Pannipara, A. G. Al-Sehemi, Y. Xia. Bimetallic metal-organic framework derived 3d hierarchical NiO/Co_3O_4/C hollow microspheres on biodegradable garbage bag for sensitive, selective, and flexible enzyme-free electrochemical glucose detection. Chem. Eng. J. **430**, 133157 (2022). https://doi.org/10.1016/j.cej.2021.133157

[S18] Y. Zhang, D. Zhao, W. Zhu, W. Zhang, Z. Yue et al., Engineering multi-stage nickel oxide rod-on-sheet nanoarrays on Ni foam: A superior catalytic electrode for ultrahigh-performance electrochemical sensing of glucose. Sens. Actuators B Chem. **255**, 416-423 (2018). https://doi.org/10.1016/j.snb.2017.08.078

[S19] V. Myndrul, E. Coy, N. Babayevska, V. Zahorodna, V. Balitskyi et al., MXene nanoflakes decorating ZnO tetrapods for enhanced performance of skin-attachable stretchable enzymatic electrochemical glucose sensor. Biosens. Bioelectron. **207**, 114141 (2022). https://doi.org/10.1016/j.bios.2022.114141

[S20] T. Liang, L. Zou, X. Guo, X. Ma, C. Zhang et al., Rising mesopores to realize direct electrochemistry of glucose oxidase toward highly sensitive detection of glucose. Adv. Funct. Mater. **29**(44), 1903026 (2019). https://doi.org/10.1002/adfm.201903026
